# Modification of Transition Metal Dichalcogenide Interlayer Interaction and Charge Transfer Through Transient Photoexcitation and Lithiation

**DOI:** 10.1002/smll.202506735

**Published:** 2025-09-18

**Authors:** Robert Haverkamp, Nomi L.A.N. Sorgenfrei, Alexander Föhlisch

**Affiliations:** ^1^ Institute for Methods and Instrumentation for Synchrotron Radiation Research Helmholtz‐Zentrum Berlin für Materialien und Energie GmbH Albert‐Einstein‐Straße 15 12489 Berlin Germany; ^2^ Institute of Physics and Astronomy University of Potsdam Karl‐Liebknecht‐Straße 24/25 14476 Potsdam Germany

**Keywords:** charge density wave, directional charge transfer, intercalation, interlayer interaction, transition metal dichalcogenide

## Abstract

The interlayer interaction mechanisms and strengths prevailing in the van der Waals gap of transition metal dichalcogenides and 2D materials in general critically influence their inherent properties and charge transfer efficiency. Through femtosecond photoexcitation, the phase‐dependent interlayer interaction strength in prototypical charge density wave‐ coupled 1T‐TaS_2_ and van der Waals‐ coupled 2H‐MoS_2_, as well as 1T‐MoS_2_ is transiently modified and compared to 1T‐Li_x_MoS_2_ reached by lithium intercalation. Sub‐femtosecond directional charge transfer times are detected through sulphur L_1_L_2,3_M_1,2,3_ core‐hole clock spectroscopy. In 1T‐TaS_2_, all‐optical modification of the charge density wave commensurability and long‐range charge modulation results in a reduced interlayer interaction, evident by a decelerated interlayer charge transfer. For 2H‐MoS_2_ transitioning to 1T‐MoS_2_, despite the photo‐induced structural modifications, the local bonding arrangement remains similar, preserving equivalent interlayer charge transfer rates. In contrast, the Li^+^ intercalation‐induced phase transition and formation of 1T‐Li_x_MoS_2_ leads to an accelerated interlayer charge transfer through a strong Coulomb interaction between the intercalated Li^+^‐ions and the charge‐accumulated 1T‐MoS_2_ crystal lattice, while the intralayer charge transfer remains unaltered. The formed electrical dipole moment within the van der Waals gap causes the opening of an efficient interlayer charge delocalization pathway through an intercalation‐mediated charge separation coupling.

## Graphical abstract

Directional charge transfer efficiency depends on the interlayer interaction strength. Femtosecond photoexcitation and Li+ intercalation reveal an invariance of intra‐ and interlayer interaction to the local bonding arrangement in van der Waals‐coupled MoS2 and charge density wave‐coupled TaS2, representative of the class of transition metal dichalcogenides. In contrast, a significant enhancement through charge imbalance states and Coulomb interactions is found.

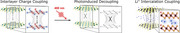

## 1 Introduction

Layered transition metal dichalcogenides (TMDs) possess highly anisotropic bonding characteristics with strong intralayer covalent/ionic bonding and comparably weak cohesive interlayer van der Waals (vdW) interaction, due to correlated fluctuating dipoles in adjacent layers. Constituent monolayers consist of a central plane of transition metal cations (such as Mo, Ta, or Ti) between two planes of chalcogen anions (S, Se, or Te) with the general stoichiometry of MX_2_.^[^ ^1^ ^]^ Distinct coordination environments of the transition metal atoms within the monolayers and their stacking sequence lead to versatile and specific crystallographic symmetries for the same chemical entity (i.e., polymorphs). The two most characteristic polymorphs are distinguished by either trigonal prismatic (2H) or octahedral (1T) coordination of the transition metal atoms.^[^ ^1^, ^2^ ^]^

Despite the weak and inherently non‐local vdW interlayer interaction, the interaction between individual layers induces phenomena that are absent in monolayers due to quantum confinement effects. This includes a dramatic layer‐dependent evolution of the electronic band structure due to interlayer hybridization, i.e., the coupling of interlayer electronic states^[^ ^3^, ^4^, ^5^ ^]^ which significantly affects the inherent properties of TMDs. Therefore, the modulation of the interlayer interaction strength and mechanisms provides an additional dimension to tailor the properties of TMDs^[^ ^6^, ^7^, ^8^ ^]^ and optimize their functionality, particularly in the field of optoelectronics based on the efficient interlayer charge transfer.^[^ ^9^, ^10^, ^11^, ^12^, ^13^, ^14^ ^]^ As the interlayer charge transfer (CT) pathways and rates are ultimately sensitive to the coupling of interlayer electronic states, CT dynamics constitute a direct measure of the interlayer interaction mechanisms and strength. The inherent dependence of the CT processes on the electronic structure,^[^ ^15^, ^16^ ^]^ and in turn the dependence of the electronic structure on the intra‐ and interlayer interactions, makes the CT characteristics sensitive to both the intralayer interaction and the interlayer interaction. An effective experimental quantification of the interlayer interaction strength by determining sub‐femtosecond CT times with both element and orbital specificity is possible by the core‐hole clock (CHC) method.^[^ ^17^, ^18^ ^]^ The viability of the technique in quantifying static in‐plane and out‐of‐plane CT processes and correlating their efficiency, respectively their speed, to the strength and mechanisms of intra‐ and interlayer interaction has been successfully utilized already. This includes several layered TMDs,^[^ ^19^, ^20^, ^21^ ^]^ TMD based heterostructures^[^ ^22^, ^23^, ^24^ ^]^ and intercalated TMD systems.^[^ ^25^, ^26^ ^]^

Despite the similar crystallographic structure in the class of layered TMDs, the interlayer interaction strengths and mechanisms prevailing in the vdW gap vary significantly. In 1T‐TaS_2_, depending on the temperature‐mediated commensurability of the charge density wave (CDW) system with the underlying crystal lattice, a variable interlayer interaction strength was revealed, evident by a diverging CT perpendicular to the crystal layers by a factor of ≈2.^[^ ^19^ ^]^ An even more pronounced acceleration of the charge delocalization in the interlayer region by a factor of ≈3.7 has been detected upon Li^+^‐ion adsorption/intercalation of 2H‐MoS_2_.^[^ ^25^ ^]^ The Li^+^ intercalation reaction is mediated by a charge donation from the Li^+^‐ions to the semiconducting 2H‐MoS_2_ conduction band, inducing a transition to the metallic 1T phase and the formation of 1T‐Li_x_MoS_2_.^[^ ^27^ ^]^ However, the nature and dependencies of the accelerated interlayer CT in 1T‐Li_x_MoS_2_ are not fully assessed, due to the interdependence of the intercalation‐ and the phase transition process. It is uncertain whether this results direct from the semiconducting 2H to metallic 1T phase transition, namely conduction band filling and lattice symmetry variations, or from Li^+^ intercalation‐induced modification of the local bonding environment and an increased free‐carrier concentration in the crystal lattice.

Both the CDW transition in 1T‐TaS_2_ and the semiconducting 2H to metallic 1T phase transition in MoS_2_ are not exclusively mediated via thermal control, respectively Li^+^ intercalation. Equivalent phase transitions can be induced temporarily by optical excitation in both 1T‐TaS_2_^[^ ^28^ ^]^ and MoS_2_.^[^ ^29^ ^]^ By combining the CHC method with optical excitation, transient phase‐dependent CT dynamics in a photoexcited state are investigated for the first time. This extends the application of the CHC method beyond the measurement of static CT characteristics and establishes a foundation for future studies. The validity of measuring dynamic CT characteristics and their use for quantifying the all‐optical modulation of interlayer interaction is demonstrated by means of the established case of the prototypical CDW system in 1T‐TaS_2_. This approach will allow to quantify the chemically pure, phase‐dependent CT dynamics in MoS_2_ to decouple the influence of Li^+^ adsorption/intercalation from the effects originating from the semiconducting 2H to metallic 1T phase transition. Ultimately, this will clarify the origin and mechanism of the significantly increased interlayer interaction in 1T‐Li_x_MoS_2_.

## 2 Results and Discussion

**Figure 1:**
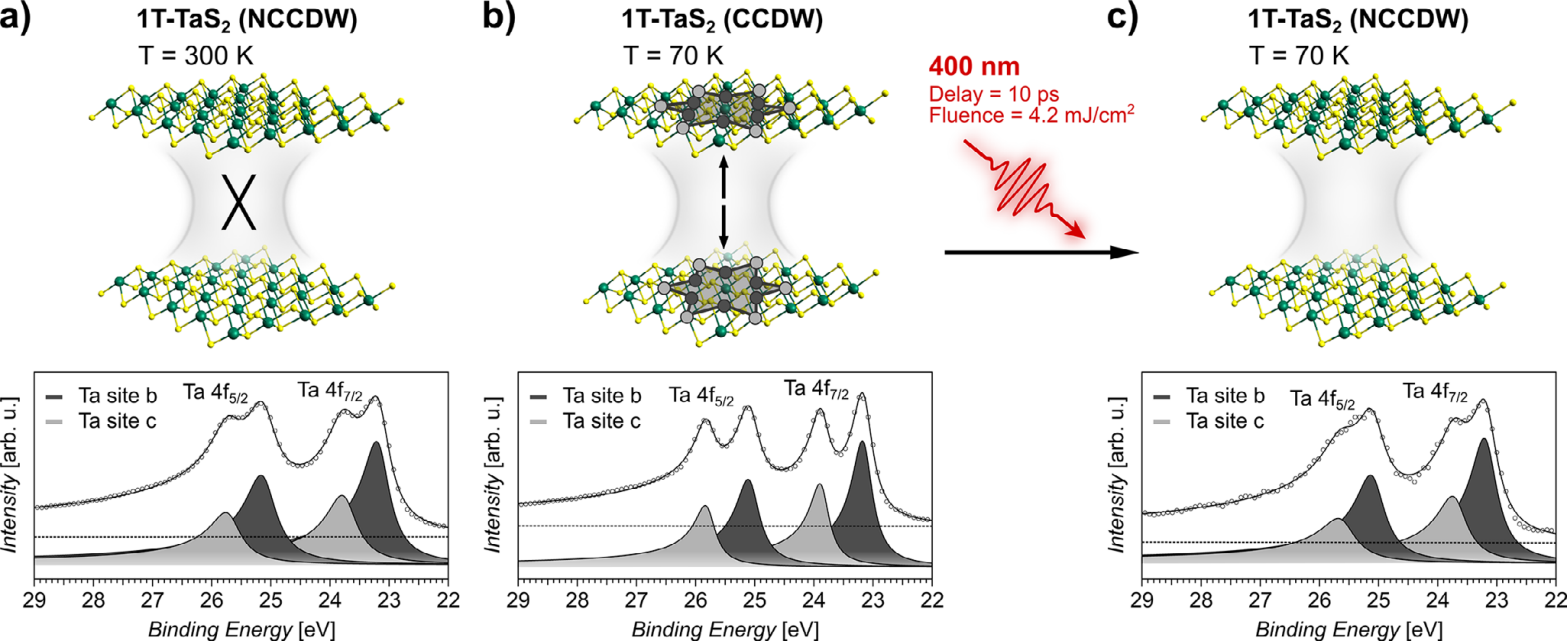
Schematically illustrated interlayer interaction of the CDW dichalcogenide 1T‐TaS_2_ in the NCCDW phase at 300 K (a), the CCDW phase at 70 K (b) and the NCCDW phase reached by 400 nm photoexcitation of the CCDW phase (c), together with the corresponding Ta 4f core‐level spectra. The known reduced interlayer interaction in the 300 K NCCDW phase is indicated by a cross. Charge interaction in the CCDW phase is indicated by arrows. Ta and S atoms are depicted by green and yellow spheres, respectively. The fits (solid curve) to the spin‐orbit split Ta 4f_5/2_ and Ta 4f_7/2_ core‐levels are obtained by a linear background subtraction (dashed line) and, accounting for the inequivalent charge densities at the Ta site b and Ta site c in the star‐shaped clusters, respectively a pair of Doniach‐Šunjić^[^ ^31^ ^]^ curves. The Ta site a contribution is not resolved sufficiently to be analyzed reliably.

The CDW formation in 1T‐TaS_2_ is defined by a simultaneous periodic modulation of the electron density into a wave structure, coupled with a periodic distortion of the crystal lattice. Depending on the commensurability, that is, the alignment between the CDW wavelength and the crystal lattice constant, 1T‐TaS_2_ exhibits a sequence of CDW phases as a function of temperature: the low‐temperature commensurate CDW (CCDW, T < 183 K), the nearly commensurate CDW (NCCDW, 183 K < T < 352 K) and the high‐temperature incommensurate CDW (ICCDW, 352 K < T < 543 K).^[^ ^30^ ^]^ The distinct temperature‐mediated CDW phases differ in their interlayer interaction strength, while the photo‐induced NCCDW phase is unexplored so far, and can be distinguished via the site‐selectivity of Ta 4f core‐level X‐ray photoelectron spectroscopy (XPS) as shown in **Figure** **1**.

The CCDW phase (Figure 1b) is characterized by a stable, well‐defined periodicity, forming 13‐Ta atom clusters in a star‐shaped arrangement where 12 Ta atoms are radially displaced toward the central Ta atom. This results in three inequivalent charge densities at the Ta sites. In contrast, S atoms are displaced primarily in the out‐of‐plane direction, with five inequivalent S sites.^[^ ^32^, ^33^ ^]^ The inequivalent local charge densities result in a distinct core‐hole screening. This is reflected most clearly in the photoemission spectra of the spin‐orbit split Ta 4f doublet (Δ_*SO*_ = 1.92 eV), which features a further well resolved CDW‐induced splitting (Δ_*CDW*_ = 0.72 eV), corresponding to the six inner (Ta site b) and the six outer (Ta site c) Ta atoms of the star‐shaped clusters.^[^ ^34^ ^]^ In response to the high degree of long‐range intra‐ and interlayer CDW ordering,^[^ ^35^ ^]^ a pronounced interaction between adjacent layers was revealed through the directional CT in 1T‐TaS_2_.^[^ ^19^ ^]^

In the NCCDW phase (Figure 1a), the temperature‐mediated gradual reduction of the coherence between the CDW and the crystal lattice leads to the coexistence of commensurate clusters separated by discommensurate domains. The coexistence of commensurate and discommensurate domains is well reflected in the shallow Ta 4f core‐levels. While the spin‐orbit splitting remained nearly unaffected (Δ_*SO*_ = 1.95 eV), a decreased CDW‐induced splitting (Δ_*CDW*_ = 0.58 eV) is evident for the reduced commensurability.^[^ ^34^ ^]^ Further details about the temperature‐induced NCCDW‐CCDW phase transition and the applied fitting procedure are given in the Supporting Information (Figure S1, Supporting Information). The loss of long‐range intra‐ and interlayer CDW ordering^[^ ^35^ ^]^ also leads to a drastically decreased interlayer interaction, evident by a decelerated CT perpendicular to the crystal layers by a factor of ≈2 in comparison to the CCDW phase.^[^ ^19^ ^]^

Besides thermal control to equilibrate the NCCDW phase, it can also be reached equivalently in a non‐equilibrium state by optical excitation.^[^ ^28^ ^]^ A prompt loss of long‐range charge‐ and lattice coherence initiated by 400 nm photoexcitation leads to an all‐optical phase transition from the highly ordered CCDW phase to the reduced ordering in the NCCDW phase (Figure 1c). The selectivity of time‐resolved XPS allows to directly monitor the transient melting and reequilibration process of the CDW commensurability. Identical to the thermally controlled NCCDW state, the Ta 4f core‐level signatures feature an unaffected spin‐orbit splitting (Δ_*SO*_ = 1.91 eV), and a reduced CDW‐induced splitting (Δ_*CDW*_ = 0.52 eV) in agreement with established values.^[^ ^28^ ^]^ Details about the applied fitting procedure are given in the Figure S1 (Supporting Information). Equivalent to the thermally controlled equilibrium states, the CT dynamics will allow to deduce the thus far unexplored interlayer interaction mechanism and strength in the photo‐induced transient NCCDW phase.

**Figure 2:**
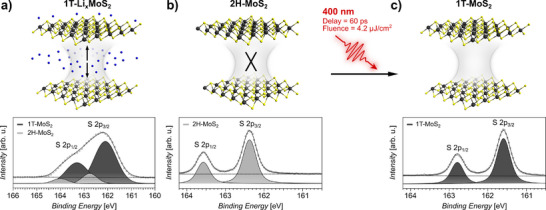
Schematically illustrated interlayer interaction of lithiated 1T‐Li_x_MoS_2_ (a), 2H‐MoS_2_ (b) and 1T‐MoS_2_ reached by 400 nm photoexcitation of 2H‐MoS_2_ (c), together with the respective S 2p core‐level spectra. The known absence of interlayer interaction for vdW coupled 2H‐MoS_2_ is indicated by a cross. Charge interaction in 1T‐Li_x_MoS_2_ is indicated by arrows. Mo, S, and Li atoms are depicted by grey, yellow and blue spheres, respectively. The fits (solid curve) to the S 2p spectra are obtained by a linear background subtraction (dashed line) and a pair of spin‐orbit split Voigt curves for contributions from the 1T and the 2H phase, respectively.

Depending on the polymorphic phase of MoS_2_, the atomic arrangement and the electronic structure differ significantly, with no CDW ordering in any phase. The naturally occurring 2H allotrope is semiconductive with a layer‐dependent bandgap and possesses a hexagonal crystal structure.^[^ ^36^ ^]^ The 1T allotrope is metallic with the Fermi level within the Mo d‐band and possesses a trigonal crystal structure.^[^ ^37^ ^]^ Charge doping reduces the stability of the 2H phase and leads to the transition to the thereafter more stable 1T phase.^[^ ^38^ ^]^ The increased charge density and redistribution of valence states is sensitively reflected in the characteristic S 2p doublet binding energies, allowing to distinguish the 2H and the 1T phase. The interlayer interaction strength of the 1T phase, obtained through Li^+^ intercalation‐induced charge doping, and the 2H phase differ significantly, while the chemically pure 1T phase, obtained through photo‐induced charge accumulation, is unexplored thus far (**Figure** **2**).

For 2H‐MoS_2_ (Figure 2b), a detailed analysis of the directional CT revealed that the weak vdW interlayer interaction in the semiconducting phase is negligible, but rather the intralayer bonding characteristics are decisive for the observed CT times.^[^ ^25^ ^]^ The semiconducting 2H phase is confirmed by means of the S 2p_1/2_ (163.6 eV) and S 2p_3/2_ (162.4 eV) core‐level binding energies.^[^ ^27^ ^]^

Lithiation of 2H‐MoS_2_ induces a transition to the 1T phase and the formation of substoichiometric 1T‐Li_x_MoS_2_^[^ ^27^ ^]^ (Figure 2a). The Li^+^‐ion adsorption/intercalation‐induced charge transfer from the Li 2s valence orbital causes a partial population and redistribution of the Mo 4d states, which drastically alters the electronic structure and the carrier density of the host lattice.^[^ ^38^ ^]^ In 1T‐Li_x_MoS_2_, the negligible vdW interlayer interaction prevailing in 2H‐MoS_2_, is found to be replaced by a significantly increased interlayer charge interaction, evident by an accelerated out‐of‐plane CT by a factor of ≈3.7.^[^ ^25^ ^]^ However, the interrelation of the intercalation‐ and the phase transition process prevents the unambiguous determination of the mechanism causing this increased interlayer interaction. The complexity arises from the co‐occurrence of the semiconductor to metal transition, the crystallographic modulation upon the 2H‐to‐1T phase transition, the increased charge carrier concentration in response to the charge donation and the interaction between the intercalated Li^+^‐ions and the charge accumulated 1T‐MoS_2_ lattice. The analysis of the S 2p_1/2_ (164 eV) and S 2p_3/2_ (162.8 eV) core‐level signatures revealed an initial ≈ 0.4 eV shift to higher binding energy, following the Li^+^‐intercalation. Additional S 2p_1/2_ (163.3 eV) and S 2p_3/2_ (162.1 eV) core‐levels shifted by ≈ 0.7 eV to lower binding energy correspond to the 1T phase.^[^ ^27^ ^]^ The quantitative evaluation of the intensity ratios confirm a dominant 1T phase‐concentration of 76 ± 5 %. An increased 1T phase concentration of 86 ± 5 % upon reduction of the X‐ray incidence angle by 30°, i.e., increasing the surface sensitivity of the measurement, confirms a predominant phase transition and formation of 1T‐Li_x_MoS_2_ within the surface layer. With the critical Li^+^ content of *x* ≈ 0.4 required to initialize the 2H‐to‐1T phase‐transition,^[^ ^39^, ^40^, ^41^ ^]^ this yields a stoichiometry of 0.34: 1 (Li: MoS_2_) within the relevant surface region. Further details about the Li^+^ deposition are given in the are given in the Experimental Section and the Supporting Information (Figures S2 and S3, Supporting Information).

Beside Li^+^‐ion adsorption/intercalation, the charge‐driven 1T phase transition can be initiated equally effective through photo‐induced charge accumulation in the surface band‐bending region.^[^ ^29^ ^]^ The controllable and fully reversible 2H‐semiconductor to 1T‐metal phase transition by means of 400 nm photoexcitation is evident by the characteristic S 2p_1/2_ (162.8 eV) and S 2p_3/2_ (161.6 eV) core‐level binding energy signatures of the 1T phase (Figure 2c). The temporal evolution of the phase transition following optical excitation and details about the fitting procedure are given in the Figure S4 (Supporting Information). The analysis of the CT dynamics in this transient photoexcited state will allow to determine the interlayer interaction strength and mechanism in the chemically pure 1T phase. Regarding the significantly accelerated CT in the interlayer region of 1T‐Li_x_MoS_2_ this will further allow to decouple the effect originating from the 2H‐1T phase transition, from the effects induced by the vicinity of Li^+^‐ions to clarify the origin of the increased interlayer interaction.

The timescale of ultrafast CT processes is derived using the CHC approach^[^ ^18^, ^42^ ^]^ at the S L_1_ absorption edge. The resonant X‐ray excitation of an S 2s core‐electron into unoccupied S 3p orbitals leads to an unstable core‐excited state with a finite lifetime serving as a temporal reference for the subsequent decay process. The decay of the core‐excited state can proceed via two competing processes, depending on whether i) the resonantly excited S 3p electron remains in an atomically localized state during the decay process,i.e., no CT occurred within the S 2s core‐hole lifetime or ii) the resonantly excited S 3p electron is decoupled from the excited atom site and delocalized within the conduction band, i.e., CT occurred within the S 2s core‐hole lifetime. Autoionization decay processes of the unstable core‐excited state before and after the occurrence of CT show different characteristic dispersive behavior. In the first case, the kinetic energy of the emitted electron possesses a linear dispersion as a function of the incident X‐ray energy (Raman‐channel). In the second case, the emitted electron possesses a constant kinetic energy (Auger‐channel). The different dispersive behavior of the two decay channels allows their unambiguous distinction and quantification by scanning the X‐ray energy across the S L_1_ absorption edge. The intensity ratio between the delocalized Auger‐channel (*I*_*Auger*_) and the localized Raman‐channel (*I*_*Raman*_) together with the S 2s core‐hole lifetime of τ_*S*2*s*_ = 0.51 ± 0.051 fs^[^ ^43^ ^]^ allows to deduce the charge transfer time (τ_*CT*_) of the resonantly excited S 3p electron within the conduction band, based on τ_*CT*_ = *I*_*Raman*_/*I*_*Auger*_ · τ_*S*2*s*_. Selection rules for the S 2s → S 3p core‐to‐bound resonant excitation provide orbital‐specificity by the used of linearly polarized X‐rays. By aligning the X‐ray electric field vector parallel or perpendicular to the sample surface, selectively in‐plane S 3p_∥_ or out‐of‐plane S 3p_⊥_ conduction band states are populated. This allows to selectively probe in‐plane (τ_∥_) and out‐of‐plane (τ_⊥_) charge transfer times. The timescale of directional CT processes reflects the degree of electronic coupling between the resonantly excited core‐electron to the conduction band state to which it is eventually transferred, thus allowing to draw conclusions about the strength and mechanism of intra‐ and interlayer interaction.

For chosen incident X‐ray energies just above the S L_1_‐edge maximum (228 eV for 2H‐MoS_2_; 226.5 eV for 1T‐TaS_2_), for resonant excitation into the conduction band minimum, where the CHC spectral signatures are reliably quantifiable, the S L_1_L_2,3_M_1,2,3_ Coster‐Kronig (CK) autoionization spectra are monitored from 30 eV to 65 eV kinetic energy. The S L_1_‐edge X‐ray absorption spectra of 1T‐TaS_2_ and 2H‐MoS_2_ are shown in in the Figure S5 (Supporting Information). The spectral decomposition and quantitative analysis of the CK autoionization decay spectra into the Raman‐channels S 2p^−1^3s^−1^3p^1^ (l) and S 2p^−1^3p^−1^3p^1^ (L) as well as the Auger‐channels S 2p^−1^3s^−1^deloc.^1^ (d) and S 2p^−1^3p^−1^deloc.^1^ (D), is described in the Figures S6 and S7 (Supporting Information). The quantitative analysis of the directional CT times will be exclusively based on the l‐ and d‐channels as these are the spectrally pure autoionization final states.^[^ ^18^ ^]^

Extending the CHC method into the time domain to study transient phase‐dependent CT dynamics in 1T‐TaS_2_ and MoS_2_ requires the coupling of exciting optical pulses with the probing X‐ray pulses. The selectivity of time‐resolved XPS allows to monitor the photo‐induced phase transition and to distinguish the phases through their characteristic core‐level signatures, analogues to the static XPS. The differing repetition rates of the 400 nm photoexcitation (6 kHz) and the X‐ray probe (1.25 MHz) allow to efficiently trace the reequilibration of 1T‐TaS_2_ and MoS_2_ following photoexcitation in 800 ns intervals (Figure S8, Supporting Information). The corresponding ≈ 166 µs interval between consecutive exciting optical pulses exceeds the respective relaxation times of the photo‐induced phase transition in both systems. This allows the simultaneous detection of the transient optically excited state and a ground‐state reference in direct succession. Only the 10 ps (1T‐TaS_2_), respectively the 60 ps (MoS_2_), time interval between the optical excitation and the probing X‐ray pulse reflects the spectrally pure photoexcited state and is considered as “pumped”. The about 166 µs time interval between the optical excitation and the probing X‐ray pulse is considered as “unpumped” and serves as the ground‐state reference.

**Figure 3:**
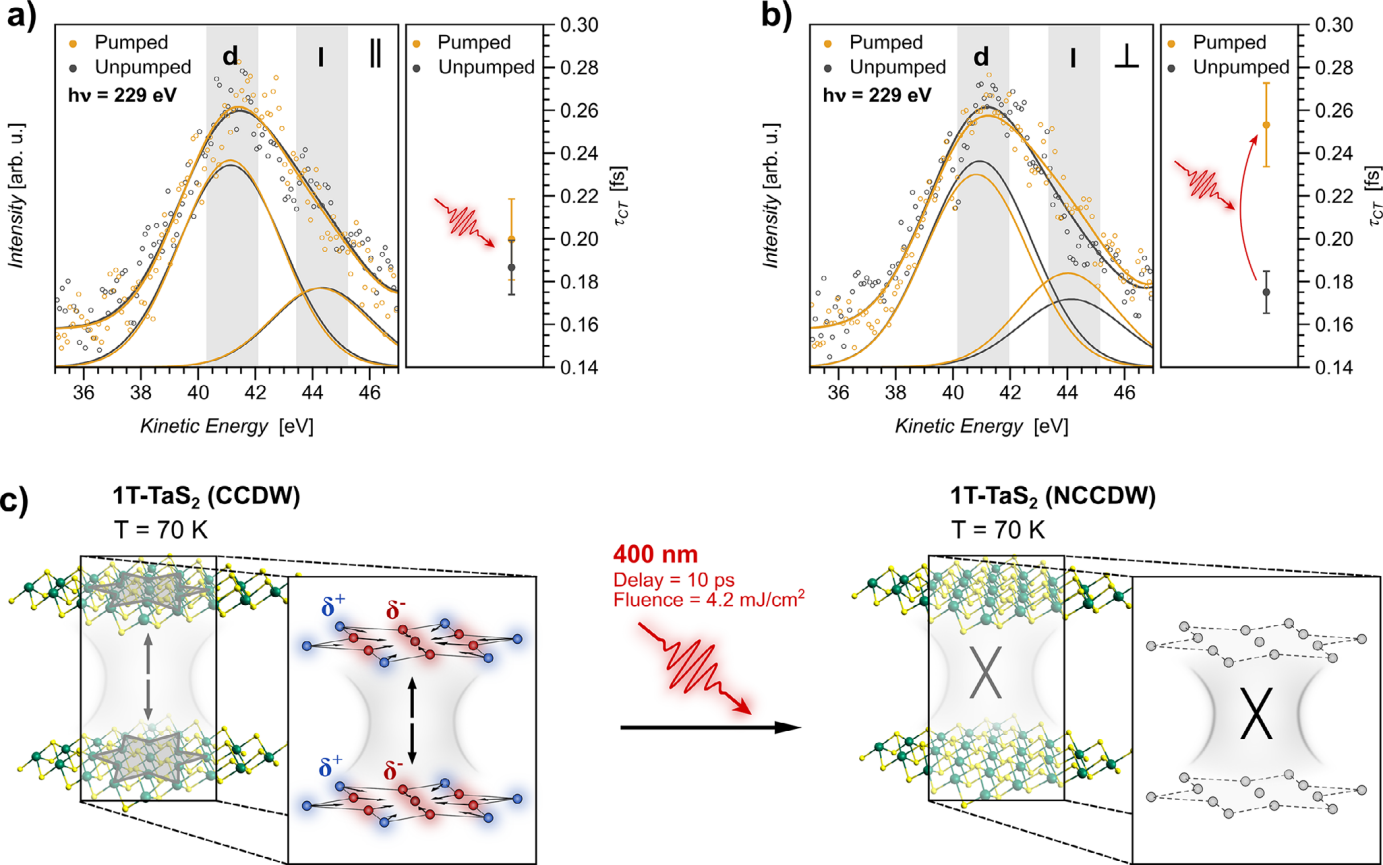
Strongly decreased interlayer charge transfer in NCCDW 1T‐TaS_2_ upon photo‐induced melting of the star‐shaped charge imbalance that is present in CCDW 1T‐TaS_2_: Exemplary S L_1_L_2,3_M_1,2,3_ CK autoionization spectra in the CCDW (unpumped) and the photoexcited NCCDW state (pumped), for in‐plane (∥) and out‐of‐plane (⊥) excitation (a and b) with the respective average CT times. The pre‐CT Raman contribution S 2p^−1^3s^−1^3p^1^ (l) and the post‐CT Auger contribution S 2p^−1^3s^−1^deloc.^1^ d) are indicated. For comparability, the spectra are shifted according to the photoexcitation‐induced kinetic energy shift of the S 2p core‐levels. Extracted τ_*CT*_ values are obtained from the weighted average of individual autoionization spectra (Figure S9, Supporting Information). Error margins result from Gaussian error propagation through the CHC analysis and the standard deviation of the weighted mean. Schematically illustrated phase transition‐ and interlayer interaction behavior of 1T‐TaS_2_ in response to 400 nm photoexcitation (c). The transient CCDW‐NCCDW phase transition manifests itself by the extensive melting of the star‐shaped charge imbalance and a resulting decrease of the interlayer interaction strength.

The central aspect of the obtained CT characteristics and the interlayer interaction mechanism in 1T‐TaS_2_ are outlined in **Figure** **3**. Exemplary photoexcitation‐dependent S L_1_L_2,3_M_1,2,3_ CK autoionization spectra from resonant in‐plane (Figure 3a) and out‐of‐plane (Figure 3b) core‐to‐bound excitation are shown in the left panel, respectively. The full range and complete set of photoexcitation‐dependent CK autoionization spectra are shown in the Figure S9 (Supporting Information). The corresponding charge delocalization times are derived from the weighted average of individual measurements and are shown in the right panel, respectively. The analysis yields directional in‐plane and out‐of‐plane charge delocalization times of τ_*CCDW*, ∥_ = 0.19 ± 0.013 fs and τ_*CCDW*, ⊥_ = 0.18 ± 0.01 fs in the CCDW ground state and τ_*NCCDW*, ∥_ = 0.2 ± 0.019 fs and τ_*NCCDW*, ⊥_ = 0.25 ± 0.02 fs in the transient NCCDW photoexcited state. Direct comparison of the local CT emanating from resonant excitation into S 3p_∥_ and S 3p_⊥_ conduction band states show that (i) in the CCDW ground state the CT proceeds without a pronounced directional dependence within the error margin of the measurement and (ii) the photo‐drive transient CCDW‐NCCDW phase transition causes a deceleration of the out‐of‐plane CT by about 40%, while the in‐plane CT remains unaffected.

The photo‐induced CCDW‐NCCDW phase transition leads from isotropic 3D CT characteristics to anisotropic 2D CT characteristics. Given the identical quasi 2D intralayer crystal structure of the CCDW and the NCCDW phase, this is ascribed to a reduced interlayer interaction. As a result of the photoexcitation, a reduction of the interlayer distance by about 0.03 Å^[^ ^44^ ^]^ is expected, assuming an instantaneous lattice response to the thermal input (Experimental Section). Accordingly, the reduction of the vdW gap leads to an increased orbital overlap between S orbitals in adjacent 1T‐TaS_2_ sheets, i.e., an increased interlayer interaction, and can not account for the observed out‐of‐plane CT deceleration. Therefore, the derived CT characteristics demonstrate that the CDW commensurability is intrinsically linked to the out‐of‐plane CT in 1T‐TaS_2_. The intrinsic mechanism, accompanying the differing interlayer interaction strength, is outlined in Figure 3c. In the CCDW phase the periodic long‐range charge modulation is concomitant with the formation of a metallic band perpendicular to the crystal layers,^[^ ^45^ ^]^ providing an efficient interlayer charge delocalization pathway and an enhanced interlayer charge interaction. The photo‐induced melting of the CDW long‐range commensurability leads to an extensive collapse of the star‐shaped Ta atom displacement and a significant charge redistribution from the periodic charge‐imbalanced state to a mostly charge neutral state in the transient NCCDW state. The associated collapse of the out‐of‐plane metallic band and the formed interlayer charge delocalization pathway, lead to a reduced interlayer charge interaction back to a mainly vdW interaction, evident by the decelerated out‐of‐plane CT from resonantly excited S 3p orbitals.

The obtained results show slight quantitative deviations from the CT characteristics in the NCCDW and CCDW equilibrium state of 1T‐TaS_2_ reached by thermal control.^[^ ^19^ ^]^ An observed overall slower CT and a lower out‐of‐plane deceleration upon the CCDW‐NCCDW phase transition might emanate from the crystal quality like vacancies/lattice mismatches or adsorbed/intercalated species and an incomplete photo‐induced CDW melting. However, the derived charge delocalization times are in excellent agreement qualitatively. Thus, it is demonstrated that the CDW‐induced interlayer charge interaction in 1T‐TaS_2_ can be controlled equally by optical excitation and confirm the validity of applying the CHC method to probe CT dynamics in a transient photoexcited state.

**Figure 4:**
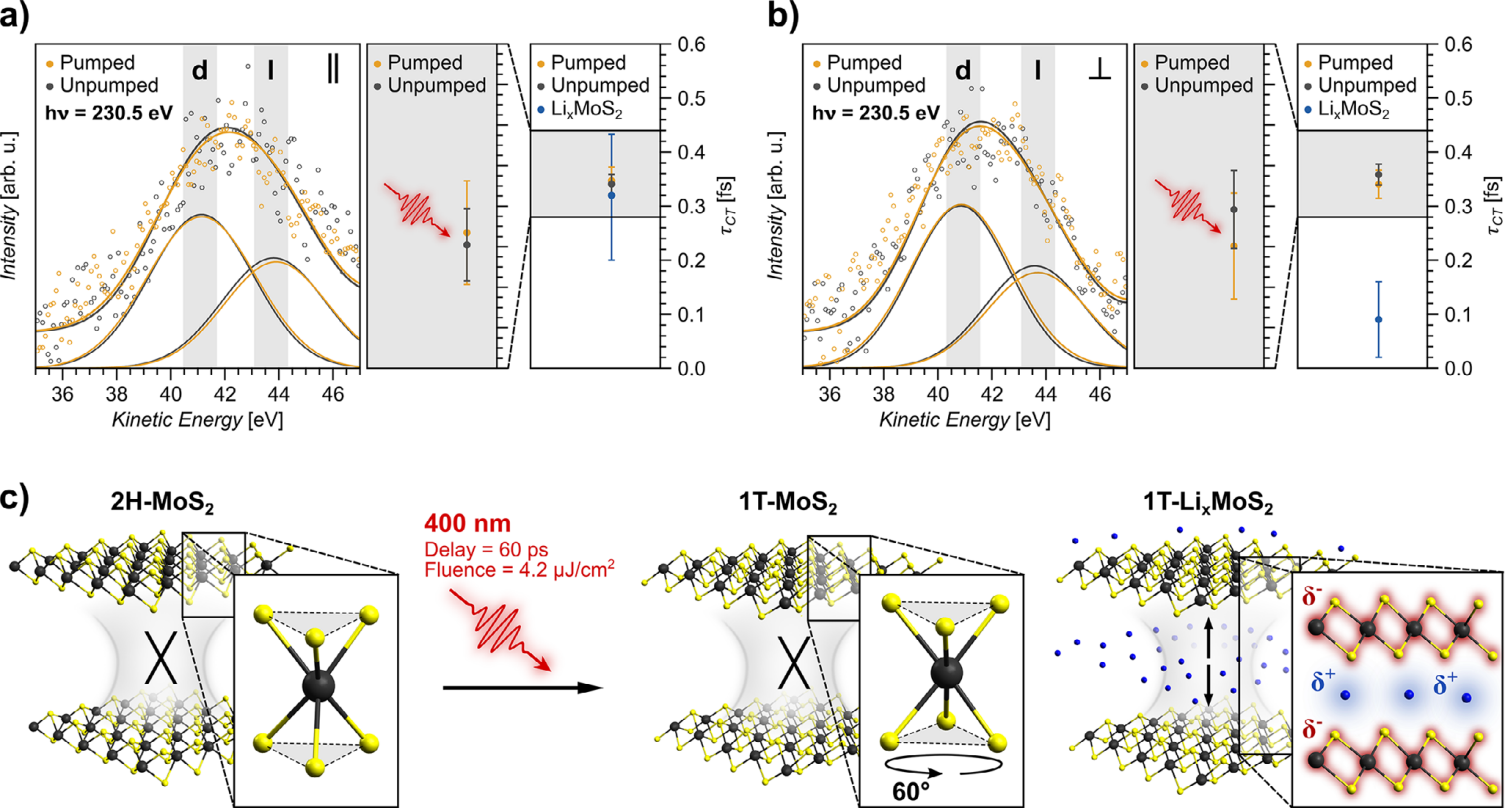
Independence of CT in 2H‐MoS_2_ and 1T‐MoS_2_ in contrast to strongly accelerated out‐of‐plane CT in 1T‐Li_x_MoS_2_ caused by charge‐mediated interlayer interaction: Exemplary S L_1_L_2,3_M_1,2,3_ CK autoionization spectra of 2H‐MoS_2_ (unpumped) and photoexcited 1T‐MoS_2_ (pumped), for in‐plane (∥) and out‐of‐plane (⊥) excitation (a and b) with the respective average CT times. The pre‐CT Raman contribution S 2p^−1^3s^−1^3p^1^ (l) and the post‐CT Auger contribution S 2p^−1^3s^−1^deloc.^1^ (d) are indicated. For comparability, the spectra are shifted according to the photoexcitation‐induced shift of the S 2p core‐levels. Extracted τ_*CT*_ values are obtained from the weighted average of individual autoionization spectra (Figure S10, Supporting Information). Error margins result from Gaussian error propagation through the CHC analysis and the standard deviation of the weighted mean. Schematically illustrated phase transition‐ and interlayer interaction behavior of MoS_2_ in response to 400 nm photoexcitation and of 1T‐Li_x_MoS_2_ (c). Structurally, the transient 2H‐1T phase transition proceeds via a 60° rotation of the S atom planes relative to each other, leaving the local charge neutrality and the interlayer interaction strength unaffected. In 1T‐Li_x_MoS_2_, the Li^+^ intercalation‐induced charge‐imbalance leads to an increased charge‐mediated interlayer interaction.

The photoexcitation‐dependent CT and interlayer interaction characteristics of MoS_2_ are depicted in **Figure** **4**. Exemplary S L_1_L_2,3_M_1,2,3_ CK autoionization spectra for the 2H ground state and the 1T photoexcited state, obtained from in‐plane (Figure 4a) and out‐of‐plane (Figure 4b) core‐to‐bound excitation are shown in the left panel, respectively. The full range and complete set of photoexcitation‐dependent CK autoionization spectra are shown in Figure S10 (Supporting Information). The corresponding CT times, derived from the weighted average of individual measurements are shown in the right panel, respectively. In the 2H ground state directional CT times of τ_2*H*, ∥_ = 0.34 ± 0.018 fs and τ_2*H*, ⊥_ = 0.36 ± 0.019 fs and in the transient photoexcited 1T state directional CT times of τ_1*T*, ∥_ = 0.35 ± 0.026 fs and τ_1*T*, ⊥_ = 0.34 ± 0.026 fs are detected in the in‐plane and the out‐of‐plane direction, respectively. It is evident that (i) highly isotropic CT characteristics prevail in both, the 2H ground state as well as the transient 1T photoexcited state and that (ii) no effect from the photoexcitation and the consequential transient 2H‐1T phase transition on the CT times is detectable within the error margins of the measurement.

The absence of CT anisotropy in regard of the macroscopically depicted highly anisotropic layered crystal structure of 2H‐MoS_2_ is accounted to the absence of any crystallographic anisotropy on the atomic scale, i.e., the local Mo‐S bonding arrangement (Figure 4c). The layer dependence of directional CT in 2H‐MoS_2_ revealed that with regard to its effect on the CT, the weak interlayer vdW interaction is negligible compared to the strong intralayer covalent bonding formed through S 3p and Mo 4d states.^[^ ^23^ ^]^ Thus, the dominant charge delocalization pathway is through intralayer Mo‐S bonds. Given the nearly rectangular S‐Mo‐S bond angle of about 82°, both the S 3p_∥_ and S 3p_⊥_ orbitals have similar projections onto the Mo‐S bonds.^[^ ^46^ ^]^ Consequently, irrespective of in‐plane or out‐of‐plane core‐to‐bound excitation, similar intralayer charge delocalization pathways are probed, which is reflected in an equal probability for CT to occur, i.e., highly isotropic CT characteristics. Within the error margins of the measurement, the derived CT times in 2H‐MoS_2_ are in agreement with established values, conforming the reproducibility of the observations.^[^ ^25^ ^]^

The local bonding arrangement as the decisive factor for the CT efficiency in 2H‐MoS_2_ is consistent with the equally isotropic CT in 1T‐MoS_2_. The absence of a detectable photoexcitation‐induced effect on the CT times demonstrates, that neither the charge accumulation nor the band structure modification upon the semiconductor‐to‐metal phase transition affect the CT characteristics. Structurally, the 2H‐1T phase transition involves a 60° rotation of the S atom planes relative to each other.^[^ ^37^ ^]^ However, this significant structural modification is leaving the local charge neutrality and the S 3p orbital projection onto the angled Mo‐S bonds unaltered. Accordingly, equal in‐plane and out‐of‐plane intralayer charge delocalization pathways are preserved as in 2H‐MoS_2_, and hence similar isotropic CT times are observed. Thus, the results demonstrate that despite the fundamental differences between the 2H phase and the 1T phase, the intralayer charge delocalization pathway through the Mo‐S bonds remains the decisive factor for the CT efficiency in 1T‐MoS_2_. Evidently, the interlayer interaction strength is unaffected by the semiconductor‐to‐metal phase transition. It should be noted, that the existence of the CDW system in 1T‐TaS_2_, although possessing a similar crystallographic structure as 1T‐MoS_2_, does not allow to transfer the same line of argumentation, given the evident impact of the CDW formation on the CT characteristics.

Ultimately, the determined CT dynamics clarify the so far unresolved origin and mechanism of the significantly increased interlayer charge interaction in 1T‐Li_x_MoS_2_ upon Li^+^ intercalation.^[^ ^25^ ^]^ The intercalation reaction causes different effects on the electronic and crystallographic structure of the 2H‐MoS_2_ host lattice. In response to the Li^+^ intercalation, the interlayer distance increases.^[^ ^47^ ^]^ Accordingly, the broadening of the vdW gap would cause a decreased interlayer interaction and can not account for the observed out‐of‐plane CT acceleration. The charge donation from the Li^+^‐ions to the 2H‐MoS_2_ host lattice causes a progressive Mo d‐band filling. This effect is identical for both the intercalation‐mediated, as well as the photo‐induced charge accumulation and can therefore also be excluded as a cause. Additionally, the charge donation causes in‐plane structural changes and band‐structure modifications upon the semiconducting 2H to the metallic 1T phase transition. Neither can account for the observed CT times in 1T‐Li_x_MoS_2_ as they are also both caused equally by the intercalation reaction and photo excitation. It is therefore concluded that the intercalated Li^+^‐ions do not simply function as charge dopants but rather induce a modification of the local charge order at the excited atom site. The intercalation‐reaction leads to the formation of an electrical dipole moment in the vdW gap and a strong Coulomb interaction between the Li^+^‐ions and the charge‐accumulated 1T‐MoS_2_ crystal lattice (Figure 4c). As a result the weak vdW interlayer interaction in 2H‐MoS_2_ is replaced by an enhanced charge‐mediated Coulomb interlayer interaction in 1T‐Li_x_MoS_2_.^[^ ^48^ ^]^ This implicates the opening of an efficient Li^+^ intercalation‐induced charge delocalization pathway. The out‐of‐plane CT acceleration by a factor of ≈ 3.7 can be considered as a lower limit for the coupling of S atoms in the 1T‐Li_x_MoS_2_ layers and the intercalated Li^+^. This is reasoned by the interlayer Li^+^ concentration gradient and the average 1T phase concentration of 86% in the surface layer. A similar acceleration of the out‐of‐plane charge delocalization by a factor of ≈3 was observed upon K^+^ intercalation of 2H‐MoS_2_.^[^ ^26^ ^]^ Equivalent to Li^+^ intercalation, the K^+^ intercalation‐reaction is mediated by a charge donation to the 2H‐MoS_2_ crystal lattice and induces the 2H‐semiconductor to 1T‐metal phase transition. The similarity between the Li^+^ and K^+^ intercalation‐reaction and their equivalent effect on the out‐of‐plane CT implies the general relevance of this finding for the interlayer interaction and the directional charge delocalization within 2D layered materials upon charged particle intercalation.

## 3 Experimental Section

### XPS Measurements

All experimental data presented in this work had been acquired at the “FEMTOSPEX Molecules and Surfaces” endstation,^[^ ^49^, ^50^ ^]^ installed at the soft X‐ray UE56/1 plane grating monochromator branch of the “FemtoSpeX” facility at Bessy II during the regular multi bunch operation mode. By means of a radio transmitter the beamline allowed an effective single pulse operation (PPRE) with variable X‐ray polarizations (linear vertical and horizontal as well as circular).^[^ ^51^ ^]^

Absolute photon energy calibration was obtained by an Argon gas cell installed in the beamline. Binding energy calibration had been obtained from a gold single crystal reference sample, installed in the measurement chamber (Figure S11, Supporting Information). The data had been collected under ambient temperature and ultra‐high vacuum (UHV) conditions at a base pressure in the measurement chamber of 2 · 10^−10^ mbar throughout the whole measurement time. The presented data had been measured under a grazing X‐ray incidence angle of 15°. For the measurements a VG Scienta‐Omicron angle‐resolved time of flight (ARTOF) electron spectrometer with a 60° acceptance angle lens system was used.^[^ ^52^ ^]^ Further, preliminary measurements had been conducted at the LowDosePES endstation at the BESSY II dipole beamline PM4.^[^ ^53^ ^]^

### Pump Laser System

The UE56/1 PGM beamline was equipped with a Ti:Sapphire laser (Legend Elite Duo, COHERENT) operated at a fundamental wavelength of 800 nm, a pulse duration of ≈ 50 fs (FWHM) and a maximum repetition rate of 6 kHz. The laser system was used for optical excitation (pump) and synchronized with the synchrotron X‐ray pulses (probe). For photoexcitation of the samples the frequency doubled 2^nd^ harmonic of the pump laser system (400 nm, FWHM ≈ 70 fs), generated by using a Beta Barium Borate (BBO) crystal, had been utilized. The isolated X‐ray pulses possess a duration of ≈50 ps (FWHM) with a repetition rate of 1.25 MHz (corresponding to about 208‐times the pump laser repetition rate), allowing to efficiently track the dynamics of the optically excited system in 800 ns steps up to a timescale of about 166 µs with a fixed pump‐probe delay. A fine adjustment of the relative delay between the laser and the X‐ray pulses was accomplished by using an optical delay stage (Newport DL325) spanning a range up to 4.4 ns.

The laser pump and X‐ray probe beams were focused onto the sample surface with an approximate spot size (horizontal · vertical) of 1.5 · 0.5 mm^2^ and 200 · 200 µm^2^, respectively. The spatial overlap between laser and X‐rays was established by means of a fluorescent screen in the sample plane and a microscope camera. Temporal overlap was established using the ARTOF to detect the arrival times of the two photon beams and a phase shifter to adjust the delay of the laser pulse relative to the X‐ray pulse. Fine adjustment of the temporal overlap was accomplished by means of the space‐charge effects on the shallow Au 4f core‐levels of a polycrystalline gold reference sample. Further details are given in the Supporting Information (Figure S12, Supporting Information). Attenuation of the laser fluence was achieved by two thin film polarizers (LAYERTEC, 400 nm, 56°, Rs > 99.8%, Rp < 2%, 800 nm, 65°, Rs > 99.8%, Rp < 2%) and additional reflective UV‐grade neutral‐density (ND) filters (Thorlabs).

### Sample Preparation

Commercially available (HQ Graphene and 2D Semiconductors) high purity (>99.995%) bulk 2H‐MoS_2_ and 1T‐TaS_2_ single crystal samples were prepared by mechanically exfoliating the crystal under UHV conditions at a pressure of about 1 · 10^−9^ mbar to ensure a pristine surface. The sample quality, structural integrity and absence of contamination is confirmed by means of in situ XPS survey measurements presented in the Figure S13 (Supporting Information).

Cooling of the 1T‐TaS_2_ samples with liquid nitrogen down to 70 K was achieved using a Janis ST‐400 cryostat. For the photo‐induced phase transition in 1T‐TaS_2_ and 2H‐MoS_2_, laser fluences of 4.2 mJcm^−2^ and 4.2 mJcm^−2^ are used, respectively. With a volumetric heat capacity of $c_{{\mathrm{TaS}}_{2}}$ = 1.52 Jcm^−3^K^−1^ and $c_{{\mathrm{MoS}}_{2}}$ = 1.94 Jcm^−3^K^−1^, this results in a temporary temperature increase of $\Delta T_{TaS_{2}}\approx 187K$ and $\Delta T_{MoS_{2}}\approx 0.2K$, respectively. A controlled in situ lithium adsorption/intercalation was achieved by thermal evaporation from a wire shaped Li dispenser (SAES Getters) facing the cleaved 2H‐MoS_2_ crystal surface at a distance of around 4 cm. The deposition was performed by resistive heating of the dispenser with a current of 8.5 A corresponding to about 850°C and the 2H‐MoS_2_ sample at room temperature. The Li^+^ deposition was performed in a stepwise manner (10, 10, 30, and 30 min) with the Li 1s, S 2p, Mo 3d and Valence band XPS signatures monitored after each step (Figures S2 and S3,Supporting Information). Throughout the whole deposition time a maximum background pressure of about 1 · 10^−8^ mbar was maintained to successfully promote the transition to metallic 1T‐Li_x_MoS_2_.

## Conflict of Interest

The authors declare no conflict of interest.

## Author Contributions

The presented measurements were performed by R.H. and N.L.A.N.S. The data were handled and analyzed by R.H. The manuscript was written by R.H. and A.F. with input from N.L.A.N.S. The research was planned by N.L.A.N.S and R.H. and directed by A.F. All authors have been given the option to comment on the manuscript.

## Supporting information

Supporting Information

## Data Availability

The data that support the findings of this study are available from the corresponding author upon reasonable request.
