# Supplementary material for: Modification of Transition Metal Dichalcogenide Interlayer Interaction and Charge Transfer Through Transient Photoexcitation and Lithiation
Source: Small. 2025 Sep 18;21(48):e06735. doi: 10.1002/smll.202506735 (PMC12674105; doi:10.1002/smll.202506735)
Supplement: Supplementary file 1 — Supporting Information [file SMLL-21-e06735-s001.pdf]

NANO · MICRO
small

Supporting Information

for *Small*, DOI 10.1002/smll.202506735

Modification of Transition Metal Dichalcogenide Interlayer Interaction and Charge Transfer Through Transient Photoexcitation and Lithiation

Robert Haverkamp, Nomi L.A.N. Sorgenfrei and Alexander Föhlisch**

Supporting Information

Modification of Transition Metal Dichalcogenide Interlayer Interaction and Charge Transfer Through Transient Photoexcitation and Lithiation

Robert Haverkamp [1,2,*], Nomi L.A.N. Sorgenfrei [1],Alexander Föhlisch [1,2,*]

[1] Institute for Methods and Instrumentation for Synchrotron Radiation Research, Helmholtz-Zentrum Berlin für Materialien und Energie GmbH, Albert-Einstein-Straße 15, 12489 Berlin, Germany
[2] Institute of Physics and Astronomy, University of Potsdam, Karl-Liebknecht-Straße 24/25, 14476 Potsdam, Germany

[*] Corresponding authors:
Robert.Haverkamp@Helmholtz-Berlin.de;
Alexander.Foehlisch@Helmholtz-Berlin.de

Temperature-induced NCCDW to CCDW phase transition in 1T-TaS_2

During the cooling of 1T-TaS_2 from 300 K down to 70 K the Ta 4f shallow core-levels have been monitored consistently. Figure S1 shows the spectral evolution in equal temporal intervals. The spectra are fitted by a linear background subtraction and a pair of Doniach-Šunjić[1] curves for each of the spin-orbit split Ta 4f core levels with equal width to account for the inequivalent charge densities at the Ta site b and Ta site c. The Ta site a contribution is not resolved sufficiently to be analyzed reliably. From the multiplicities of the final states, an intensity ratio of 4:3 (Ta $4f_{7/2}$: Ta $4f_{5/2}$) is retained, with a spin-orbit splitting of 1.9 ± 0.05 eV. Due to the distinctly different local charge densities in the star-shaped clusters, the asymmetry parameters of the Doniach-Šunjić curves are kept identical for the signals from Ta sites b and those from Ta sites c. In the 300 K NCCDW phase, the coexistence of commensurate clusters separated by discommensurate domains is reflected in a moderate CDW-induced splitting of the Ta $4f_{5/2}$ and the Ta $4f_{7/2}$ doublet of 0.55 eV. The peak width amounts to 0.3 eV (FWHM). Upon cooling down, a gradual increase of the CDW-induced splitting and a decreasing peak width is observed. In the 70 K CCDW phase, a pronounced CDW-induced splitting of the Ta $4f_{5/2}$ and the Ta $4f_{7/2}$ doublet of 0.74 eV and an decreased peak width of 0.23 eV (FWHM) is observed.

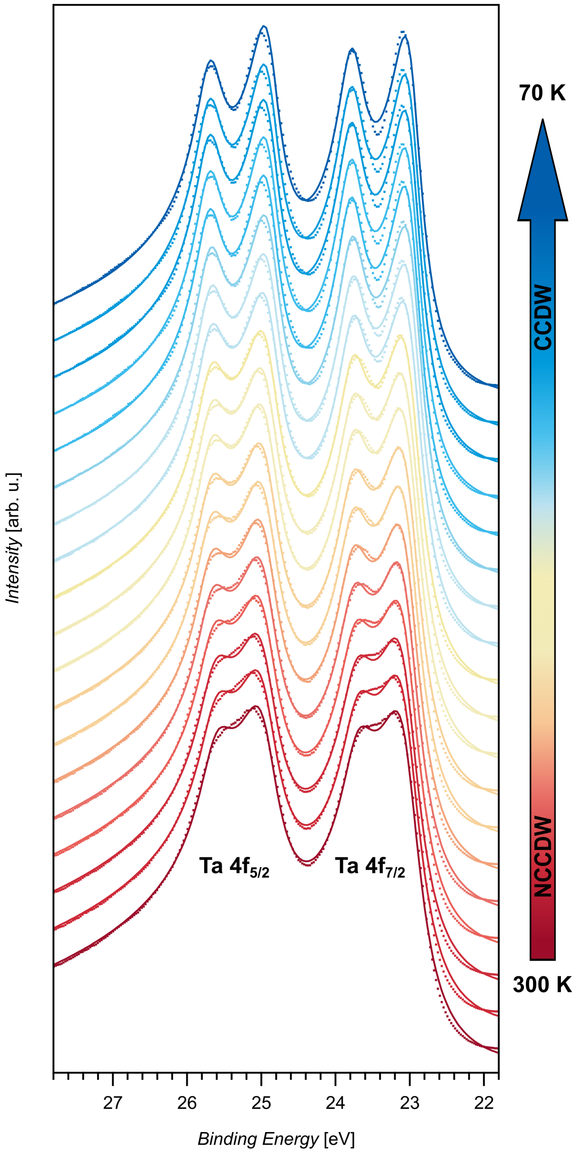

Figure S1. Spectral evolution of the spin-orbit split Ta 4f shallow core-levels in dependence of the 1T-TaS_2 temperature. Upon cooling down from 300 K to 70 K, a distinct CDW-induced splitting and a reduced width of the Ta 4f core-levels becomes apparent, indicative for the NCCDW-CCDW phase transition.

Lithium deposition onto 2H-MoS_2

By means of the Li 1s core-level signature the Li^+ deposition on the 2H-MoS_2 surface is monitored. The spectral evolution in dependence of the stepwise Li^+ deposition time is shown in Figure S2. The spectra are taken after 10, 20, 50 and 80 minutes of Li^+ deposition under otherwise unchanged experimental conditions. A gradual increase of the Li 1s signal intensity confirms the successful deposition of Li^+ onto the 2H-MoS_2 sample surface. An intermediate annealing of the sample for 30 minutes at 130°C shows no noticeable decrease of intensity, binding energy shift or broadening of the Li 1s core-level. This confirms the absence of Li^+ depletion as well as the structural and chemical integrity of the intercalation/adsorption process and the crystallographic state reached.
The fitting procedure of the Li 1s core-level involves a linear background subtraction and a Voigt peak at a binding energy of 54.9 eV. The Voigt line profile is dominated by the experimental Gaussian broadening of 0.89 eV (FWHM) over the Lorentzian life-time broadening of 0.55 eV (FWHM).

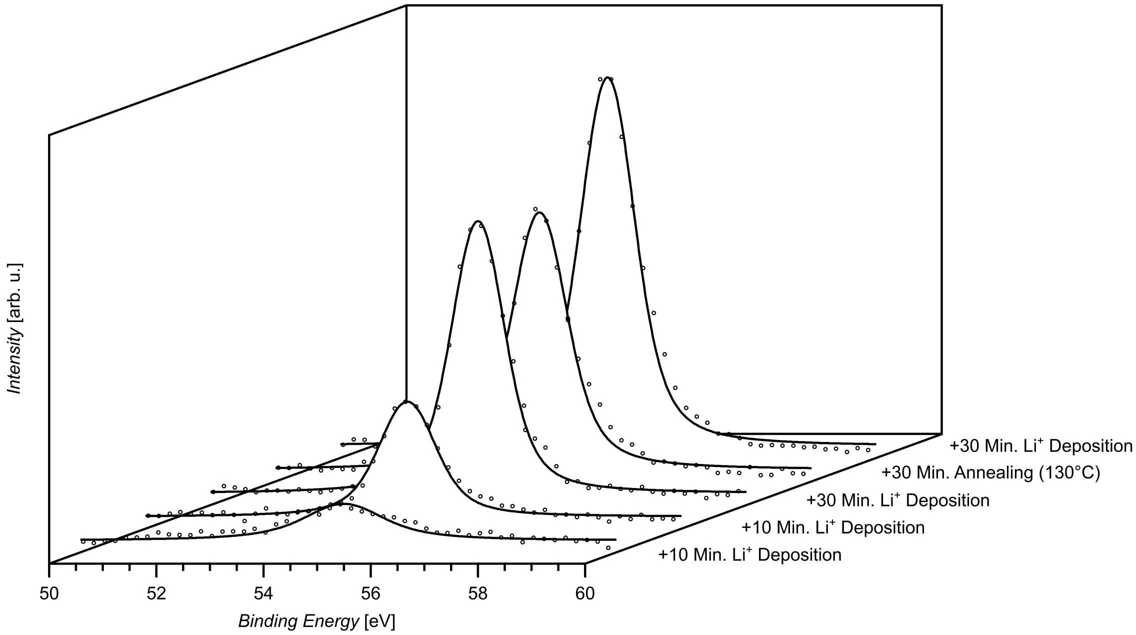

Figure S2. Spectral evolution of the Li 1s core-level signature in dependence of Li intercalation/adsorption time and sample annealing. The increasing signal intensity under otherwise unchanged experimental conditions confirms the gradual Li^+ deposition on the 2H-MoS_2 surface. An intermediate heating to 130°C does not alter the signal intensity, confirming the stability of the deposited Li^+.

Lithium intercalation-induced $2H\text{-}MoS_2$ to $1T\text{-}Li_xMoS_2$ phase transition

After 10, 20, 50 and 80 minutes of Li^+ adsorption/intercalation of $2H\text{-}MoS_2$, a set of XPS spectra consisting of a valence band scan as well as Mo 3d and S 2p core-level spectra have been recorded. The XPS spectra prior to lithiation and after the final Li^+ adsorption/intercalation step are shown in Figure S3. The fitting procedure of the S 2p and Mo 3d doublets involves a linear background subtraction and respectively a pair of Voigt peaks for the contributions of the 2H and the 1T phase with a spin-orbit splitting of 1.16 eV (S 2p) and 3.14 eV (Mo 3d). According to their physical origin, a multiplicity of 1:2 (S $2p_{1/2}$: S $2p_{3/2}$) and 2:3 (Mo $3d_{3/2}$: Mo $3d_{5/2}$) was retained.

Before Li^+ adsorption/intercalation, the S $2p_{3/2}$ and Mo $3d_{5/2}$ core-level binding energies are found at 229.7 eV and 162.45 eV, respectively with a width (FWHM) of 0.59 eV (S 2p) and 0.74 eV (Mo 3d). After 10 minutes of Li^+ adsorption/intercalation, an about 0.4 eV shift to higher binding energy of the Mo 3d and S 2p core-levels and a slightly increased width by about 0.1 eV is observed. With further lithiation an additional pair of Mo 3d and S 2p species emerge, shifted by about 0.7 eV to lower binding energy with respect to the $2H\text{-}MoS_2$ core-levels which are attributed to the 1T phase[2]. After 80 minutes of Li^+ adsorption/intercalation, the 1T core-level components are more intense than the initial 2H core-level components. The Mo 3d and S 2p species corresponding to the 1T phase have a width of 0.7 eV and 1.1 eV (FWHM), respectively.

The quantitative evaluation of the obtained intensity ratios from the S 2p and Mo 3d core levels confirmed the coexistence of the 2H and the 1T phase with a dominant 1T phase concentration of 76 ± 5 % after the final Li^+ adsorption/intercalation step. After increasing the surface sensitivity of the measurement by reducing the X-ray incidence angle by 30°, a 1T phase concentration of 86 ± 5 % is detected. A substantial spectral modification and shift of the valence band towards the Fermi energy (E_F) further verifies the successful electron donation of the Li^+-ions to the $2H\text{-}MoS_2$ lattice and the transition to the metallic 1T phase[2].

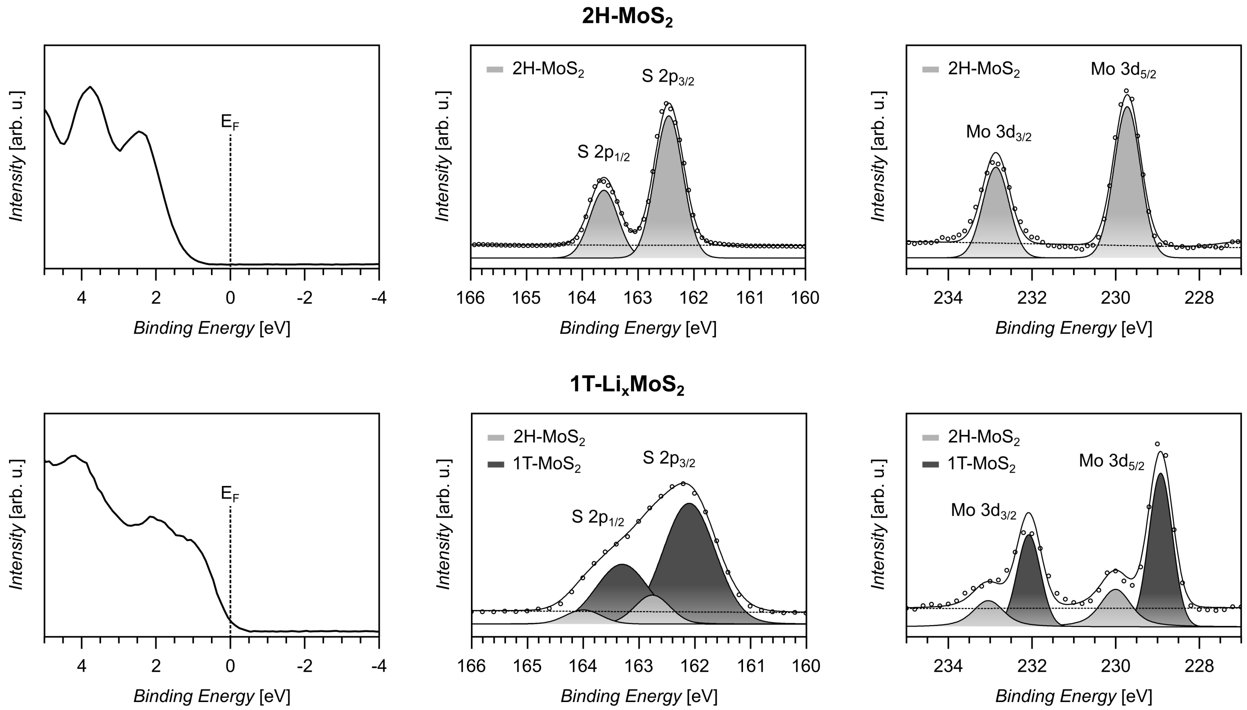

Figure S3. Valence band, S 2p and Mo 3d spectral decomposition of $2H\text{-}MoS_2$ prior to Li^+ deposition (upper panel) and of $1T\text{-}Li_xMoS_2$ after 80 minutes of Li^+ adsorption/intercalation (lower panel), confirming the successful 2H-1T phase transition. Contributions from the 2H phase and the 1T phase are indicated in light grey and dark grey, respectively.

Photo-driven 2H-semiconductor to 1T-metal phase transition in MoS_2

The sensitivity of the core-level binding energy to the chemical surrounding allows to decompose and identify semiconducting 2H-MoS_2 and metallic 1T-MoS_2 by means of the S 2p core-level multiplet. The photoexcitation-induced transient 2H-1T phase transition is illustrated in Figure S4 for different time delays between the 400 nm optical pulse with a fluence of 4.2 μJ/cm^2 and the probing X-ray pulse.

For a negative delay time of -150 ps, the characteristic S 2p binding energies of 163.4 eV (S $2p_{1/2}$) and 162.2 eV (S $2p_{3/2}$) verify the semiconducting 2H phase. For a positive delay time of +10 ps after optical excitation, the S $2p_{1/2}$ and S $2p_{3/2}$ core-levels consist of two components with similar intensity. The optical excitation generates electron-hole pairs with electrons in the conduction band accumulating in the surface layers and holes in the valence band accumulating in the bulk crystal. Due to the electron accumulation in the surface layers, an about 0.3 eV surface photovoltage shift to lower binding energy is observed compared to the 2H-MoS_2 ground state. Additionally the electron accumulation induces the phase transition from the 2H phase and stabilizes the metallic 1T phase, evident by the additional component shifted by about 0.8 eV to lower binding energy compared to the 2H-MoS_2 ground state. For a positive delay time of +100 ps the 2H-MoS_2 components have disappeared, indicating the complete phase transition to the 1T phase in the surface layers[3].

The fitting procedure to the spin-orbit split S 2p core-levels includes a constant background subtraction and respectively a pair of Voigt peaks for the contributions of the 2H and the 1T phase. The spin-orbit splitting was kept at 1.19 eV with a multiplicity of 1:2 (S $2p_{1/2}$: S $2p_{3/2}$). The Voigt line profiles are dominated by the experimental Gaussian broadening of 0.3 eV (FWHM) over the S 2p Lorentzian life-time broadening of 0.2 eV (FWHM).

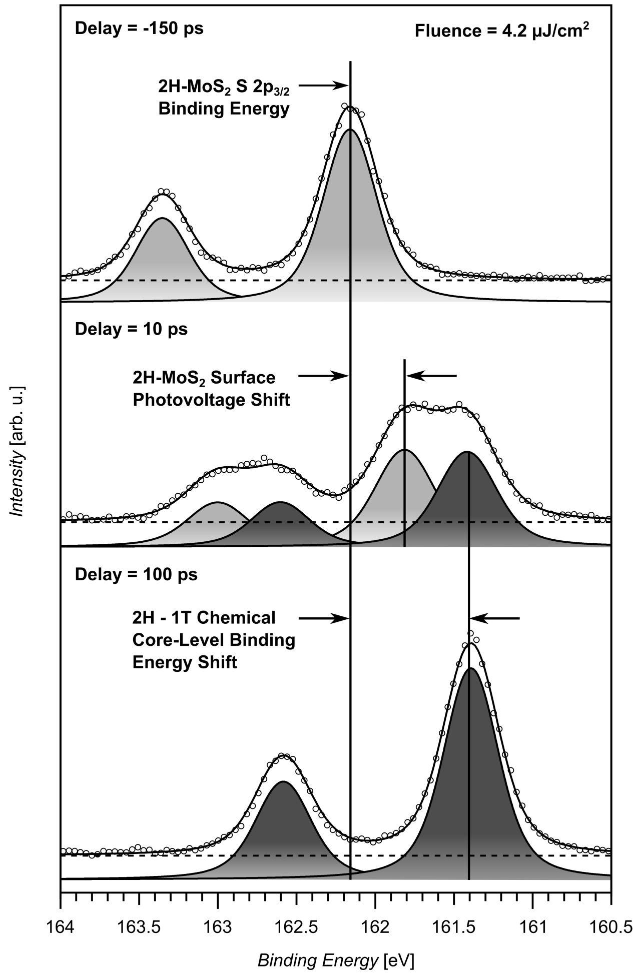

Figure S4. Photo-driven surface-layer 2H-semiconductor to 1T-metal phase transition in MoS_2. The spectral decomposition of the S $2p_{1/2}$ and S $2p_{3/2}$ core-levels in dependence of the delay time (-150 ps, 10 ps, 100 ps) allows the identification of the 2H-MoS_2 ground state, the electron accumulation induced surface photovoltage shift and of the chemically shifted 1T-MoS_2 photoexcited state.

Sulfur L_1-edge XAS of 1T-TaS_2 and 2H-MoS_2

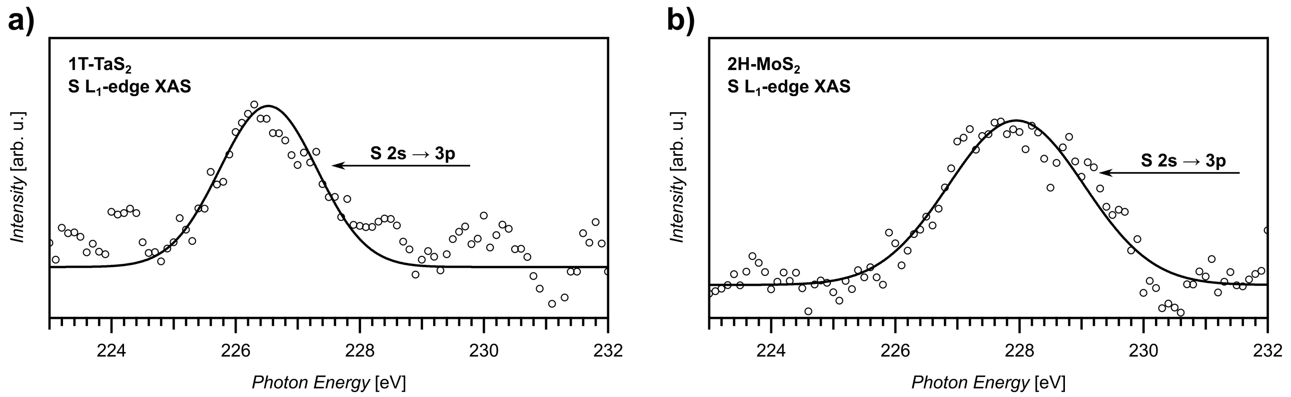

Figure S5. X-ray absorption spectra of 1T-TaS_2 (**a**) and 2H-MoS_2 (**b**) at the S L_1 absorption edge, with the S 2s → S 3p resonance maximum at 226.5 eV and 228 eV, respectively. The spectra are measured in the total electron yield (TEY) configuration using the photoelectron current and obtained by a linear background subtraction.

Spectral evolution and decomposition

The spectral decomposition is exemplary shown in Figure S6 for 1T-TaS_2 in the CCDW ground state (**a**) and for the 2H-MoS_2 ground state (**b**), respectively for incident X-ray energies below (222 eV) and above the S L_1-absorption edge maximum (229 eV and 230.5 eV). All spectral intensities are normalized to the incident X-ray flux. For photon energies below the S L_1- absorption edge, no resonant excitation occurs and the measured spectra are exclusively composed of features corresponding to the direct S 2p photoionization, shake-up processes and the spectral background. To account for the effect of inelastic scattering energy losses and secondary electron emission, the background is modeled up to the direct S 2p photoionization line by a combined Shirley background function[4] with an exponential background function. Subsequently, the direct S 2p multiplet photoionization line shape, including the corresponding shake-up features, have been shifted according to the varying incident X-ray energy and the respective photoexcitation-induced shift of the S 2p core-levels and subtracted from each spectrum. For X-ray photon energies above the S L_1-absorption edge, only the spectral components corresponding to the S $L_1L_{2,3}M_{1,2,3}$ CK autoionization decay following the S 2s $\rightarrow$ S 3p resonant excitation, remain.

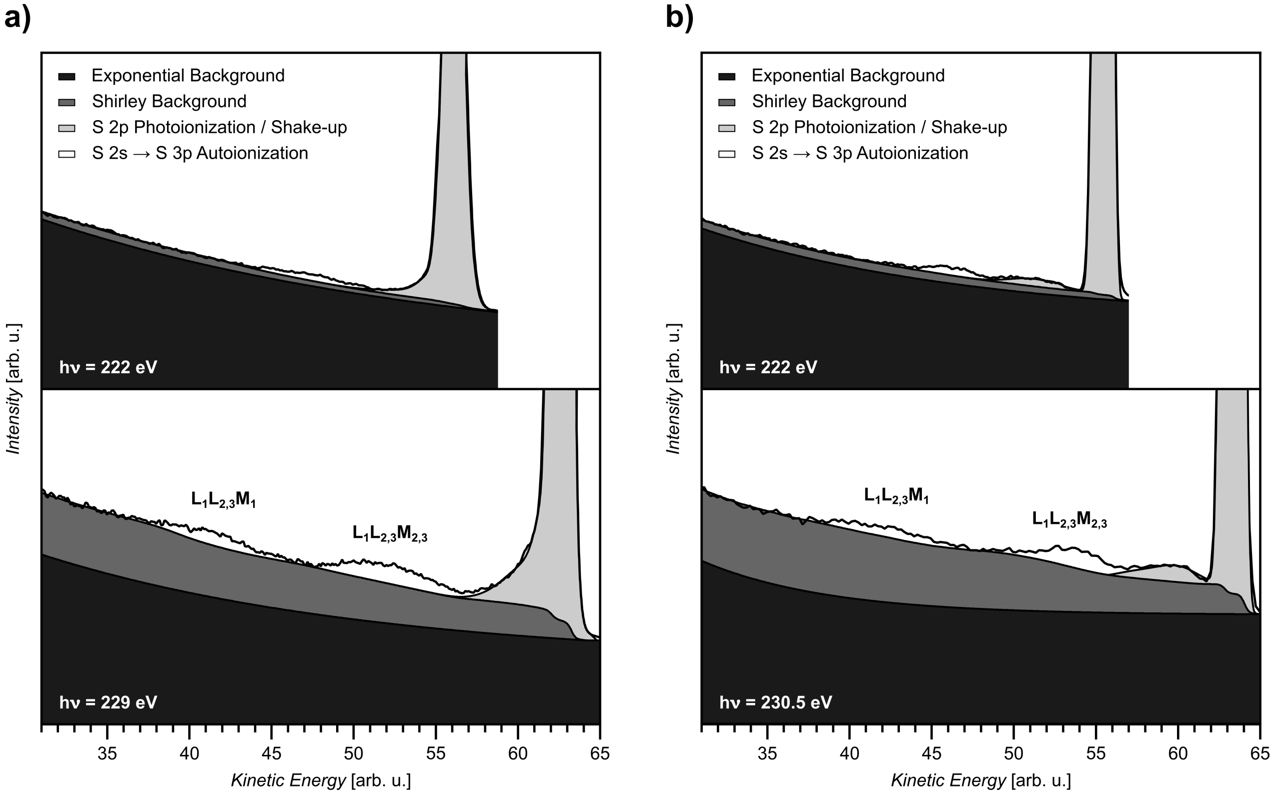

Figure S6. Exemplary spectral decomposition and evolution of the 1T-TaS_2 CCDW ground-state (**a**) and the 2H-MoS_2 ground-state (**b**) autoionization spectra, for exciting X-ray energies below (222 eV) and above (229 eV and 230.5 eV) the S L_1-absorption edge maximum. The kinetic energy region beyond the direct S 2p photoionization lines has been removed.

Quantitative $L_1L_{2,3}M_{1,2,3}$ CK autoionization decay channel analysis

To quantitatively evaluate the localized Raman- and the delocalized Auger-channel contributions to the autoionization spectra, a peak fitting routine has been used. Exemplary, the fitting procedure is shown in Figure S7 for 1T-TaS_2 in the CCDW ground state (**a**) and for 2H-MoS_2 (**b**), respectively for in-plane ($\parallel$) and out-of-plane ($\perp$) excitation above the S L_1 absorption edge maximum. The d- and l-channel contributions are depicted in red and blue, respectively. The D- and L-channel contributions are depicted in light grey and dark grey, respectively.

According to their physical origin, the charge transfer Auger-channels are kept at a constant kinetic energy of 40.9 ± 0.1 eV respectively 40.8 ± 0.1 eV (d) and 50.6 ± 0.1 eV respectively 51.1 ± 0.1 eV (D) for 1T-TaS_2 and 2H-MoS_2. The localized Raman-channels are kept at constant binding energy of 180.2 ± 0.1 eV respectively 181.8 ± 0.1 eV (l) and 170.5 ± 0.1 eV respectively 171.8 ± 0.1 eV (L) for 1T-TaS_2 and 2H-MoS_2. For the respective photoexcited states, the kinetic energy of the Auger-channels and the binding energy of the Raman-channels are shifted according to the photoexcitation-induced shift of the S 2p core-levels.

The width of the final states reached, respectively the width of the spectral channels, reflects the convolution of the natural S 2s core-hole lifetime broadening, the experimental broadening and the width of the energy levels involved in the $L_1L_{2,3}M_{1,2,3}$ CK autoionization decay process. The width of the band-like S 3s respectively S 3p states are dominating the broadening over the atomic S 2p states and the S 2s lifetime broadening. The spectral distribution of each decay channel (d, D, l, L) is approximated by a Gaussian line shape with an equal width of 4.1 ± 0.1 eV (FWHM) for 1T-TaS_2 and of 4.5 ± 0.1 eV (FWHM) for 2H-MoS_2. Only the intensities of the autoionization decay channels are kept as variable parameters.

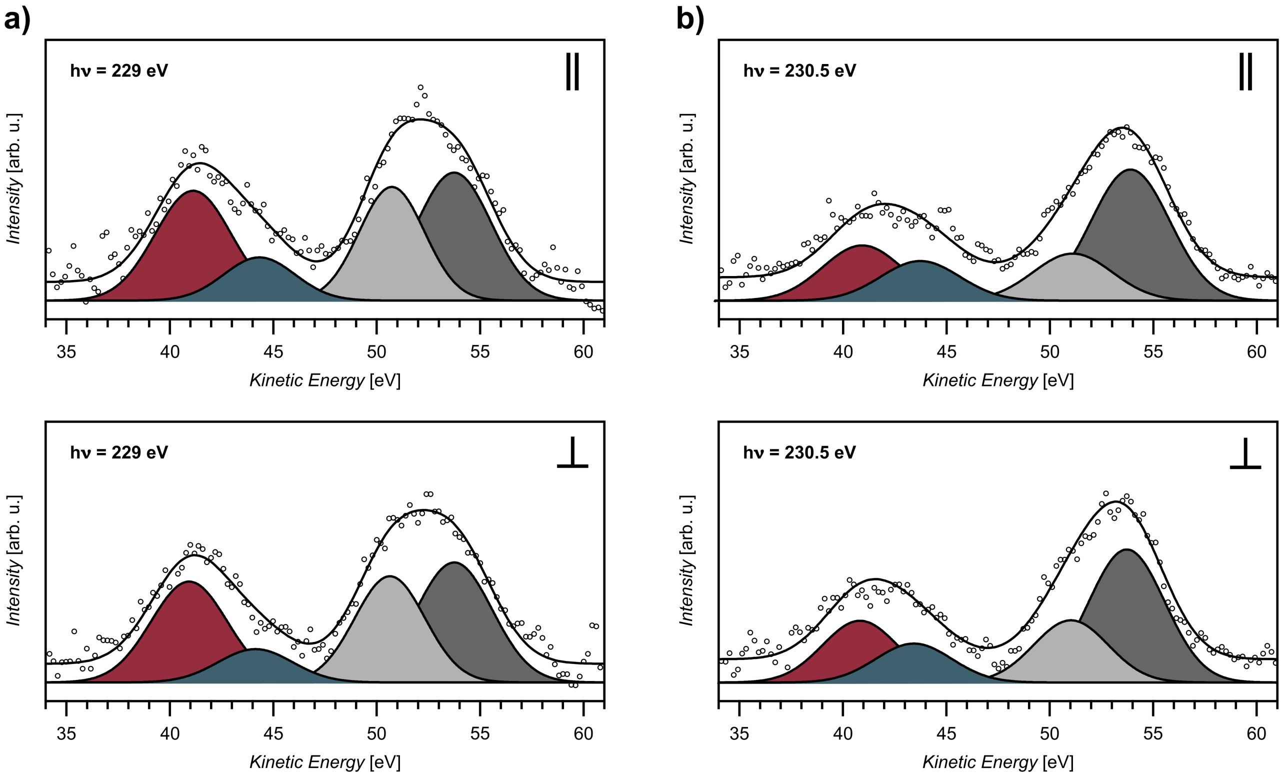

Figure S7. Fitting routine to the directional S $L_1L_{2,3}M_{1,2,3}$ CK autoionization spectra exemplary for 1T-TaS_2 in the CCDW ground state (**a**) and for 2H-MoS_2 (**b**). Excitation into S 3p orbitals in the in-plane direction ($\parallel$) as well as in the out-of-plane direction ($\perp$) are exemplarily shown for X-ray energies above the S L_1 absorption edge at $h\nu = 229$ eV (1T-TaS_2), respectively at $h\nu = 230.5$ eV (2H-MoS_2). The d- and l-channel contributions are depicted in red and blue, respectively. The D- and L-channel contributions are depicted in light grey and dark grey, respectively.

Ground-state reequilibration of 1T-TaS_2 and 1T-MoS_2

Figure S8 shows the fully reversible reequilibration of 1T-TaS_2 from the NCCDW photoexcited state to the CCDW ground state and of MoS_2 from the 1T photoexcited state to the 2H ground state. The reequilibration dynamics is monitored up to 166 μs time delay between the 400 nm optical excitation and the probing X-ray pulses with a 800 ns spacing.

The relaxation of 1T-TaS_2 from the photoexcited NCCDW state back to the CCDW ground state is confirmed through the characteristic CDW-induced splitting of the shallow Ta 4f core-levels (Figure S8**a**). After a time delay of 10 ps between the optical excitation with a fluence of 4.2 mJ/cm^2 and the probing X-rays, a prompt loss of long-range commensurability induces the transition from the CCDW to the NCCDW state, evident by a reduced CDW splitting of 0.54 eV (yellow shaded area). After 800 ns, the CDW splitting is increased again and remains within the error margins at 0.61 eV, evident for the return to the CCDW ground state (grey shaded area). The fitting procedure includes a pair of Doniach-Šunjić[1] curves for each of the spin-orbit split Ta 4f core-levels with equal width. A spin-orbit splitting between the Ta $4f_{5/2}$ and the Ta $4f_{7/2}$ doublet of 1.91 eV and a multiplicity of 3:4 is retained for all spectra. Asymmetry parameters are kept identical for the signals from Ta sites b and Ta sites c in the star-shaped clusters.

The reequilibration from the photoexcited 1T-MoS_2 state to the 2H-semiconducting ground state is monitored and verified by means of the S $2p_{3/2}$ binding energy (Figure S8**b**). After a time delay of 100 ps between the optical excitation with a fluence of 4.2 μJ/cm^2 and the probing X-ray pulses, a binding energy shift of about 750 meV indicates the transition to the 1T phase through a surface layer charge accumulation (yellow shaded area). After about 10 μs a remaining surface photovoltage binding energy shift of about 300 meV (Figure S4) indicates the relaxation of the 1T phase to the 2H phase (grey shaded area)[3]. The fitting procedure involves a pair of Gaussian peaks for the S 2p doublet with a spin-orbit splitting of 1.2 eV and a multiplicity of 1:2 ($2p_{1/2}$: $2p_{3/2}$). An equal width of 0.45 eV (FWHM) is retained for all spectra.

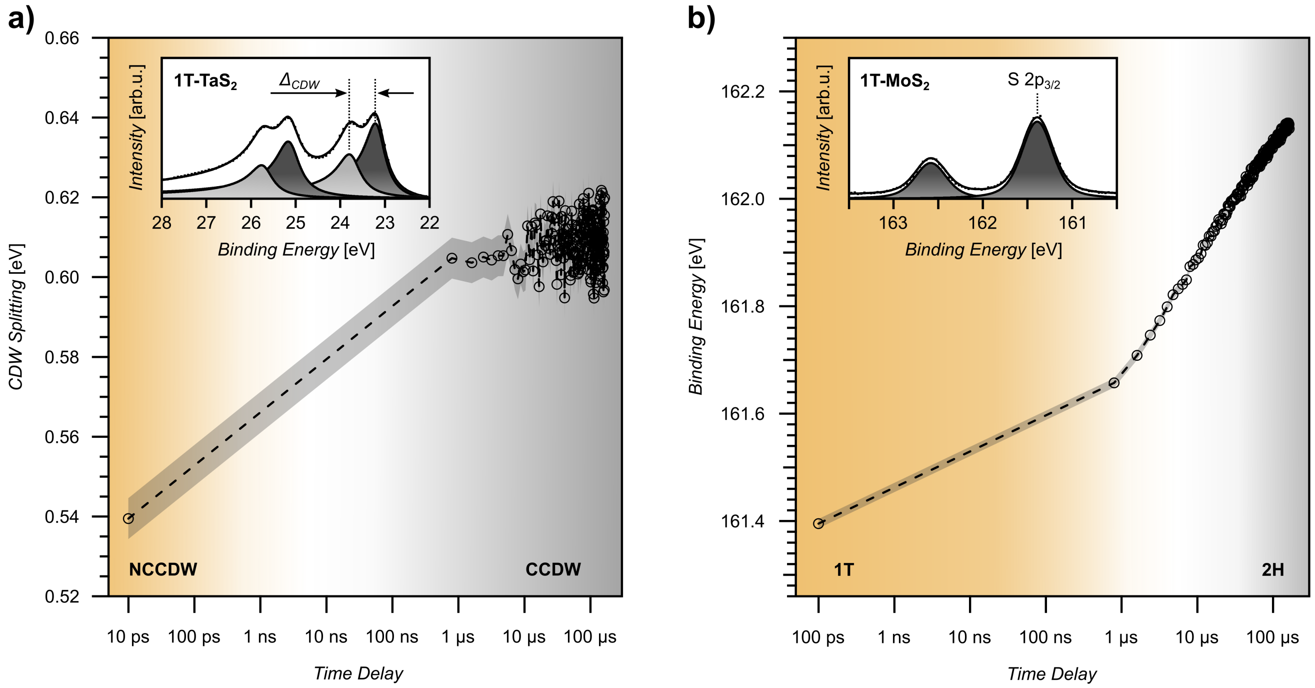

Figure S8. Reequilibration of the photo-induced CCDW-NCCDW phase transition in 1T-TaS_2 by means of the CDW-induced splitting of the shallow Ta 4f core-levels (**a**) and of the 2H-1T phase transition in MoS_2 (**b**) by means of the S $2p_{3/2}$ binding energy. The time delay between the optical excitation and the probing X-ray pulses is monitored up to 166 μs in 800 ns intervals. The photoexcited- (yellow shaded) and reequilibrated states (grey shaded) are indicated. Error margins result from the standard error mean.

Directional- and photoexcitation dependent S $L_1L_{2,3}M_{1,2,3}$ CK autoionization spectra of 1T-TaS_2

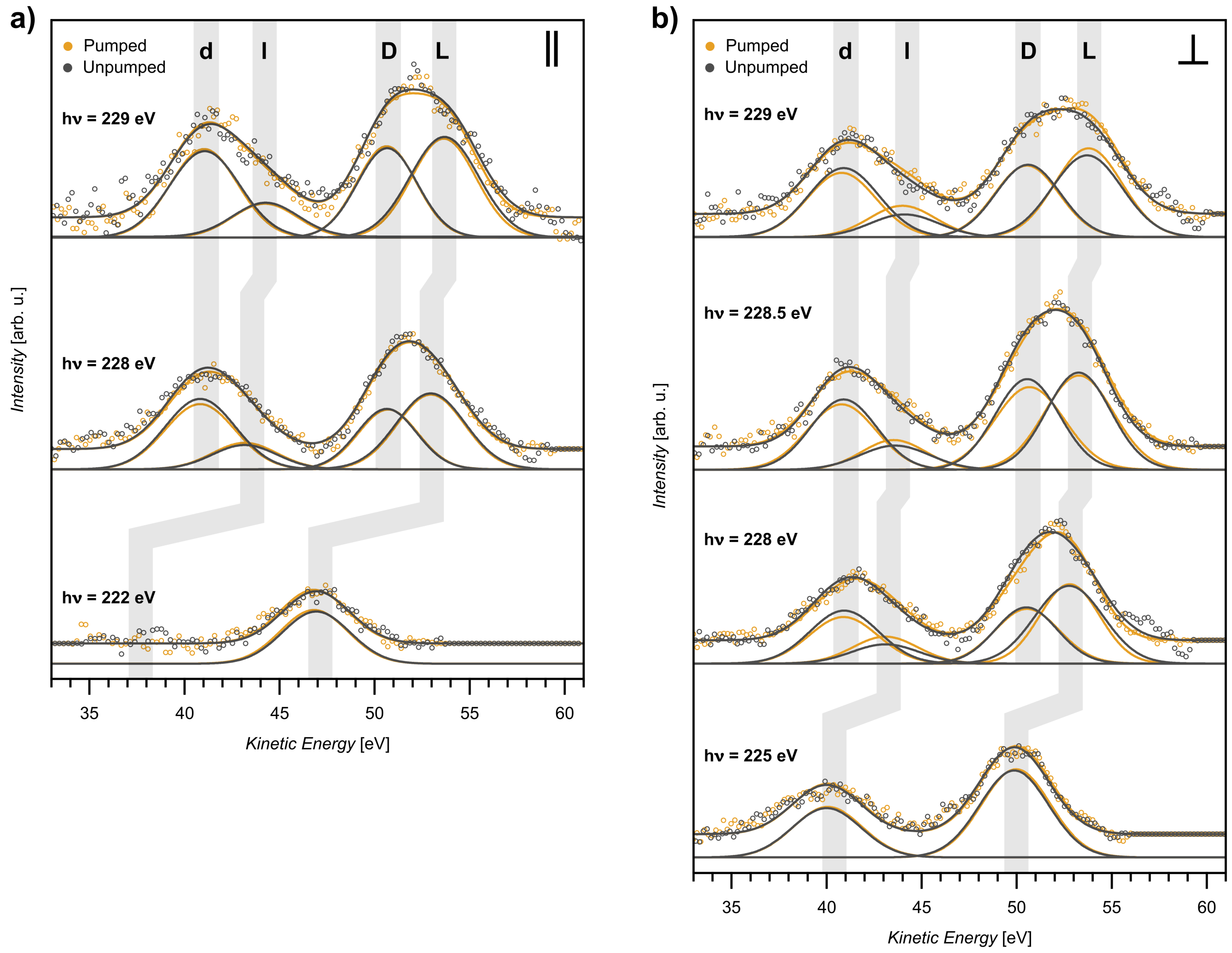

Figure S9. Directional S $L_1L_{2,3}M_{1,2,3}$ CK autoionization spectra of 1T-TaS_2 in dependence of the photon energy and optical excitation for selective preparation of S 3p excited states in the in-plane (||) direction (**a**) and in the out-of-plane (⊥) direction (**b**). The localized Raman-channels S $2p^{-1}3s^{-1}3p^{1}$ (l) and S $2p^{-1}3p^{-1}3p^{1}$ (L) as well as the delocalized Auger-channels S $2p^{-1}3s^{-1}$deloc.[1] (d) and S $2p^{-1}3p^{-1}$deloc.[1] (D) are indicated. The spectral region of the direct S 2p photoionization has been removed. For comparability, the spectra are shifted according to the photoexcitation-induced shift of the S 2p core-levels.

Directional- and photoexcitation dependent S $L_1L_{2,3}M_{1,2,3}$ CK autoionization spectra of MoS_2

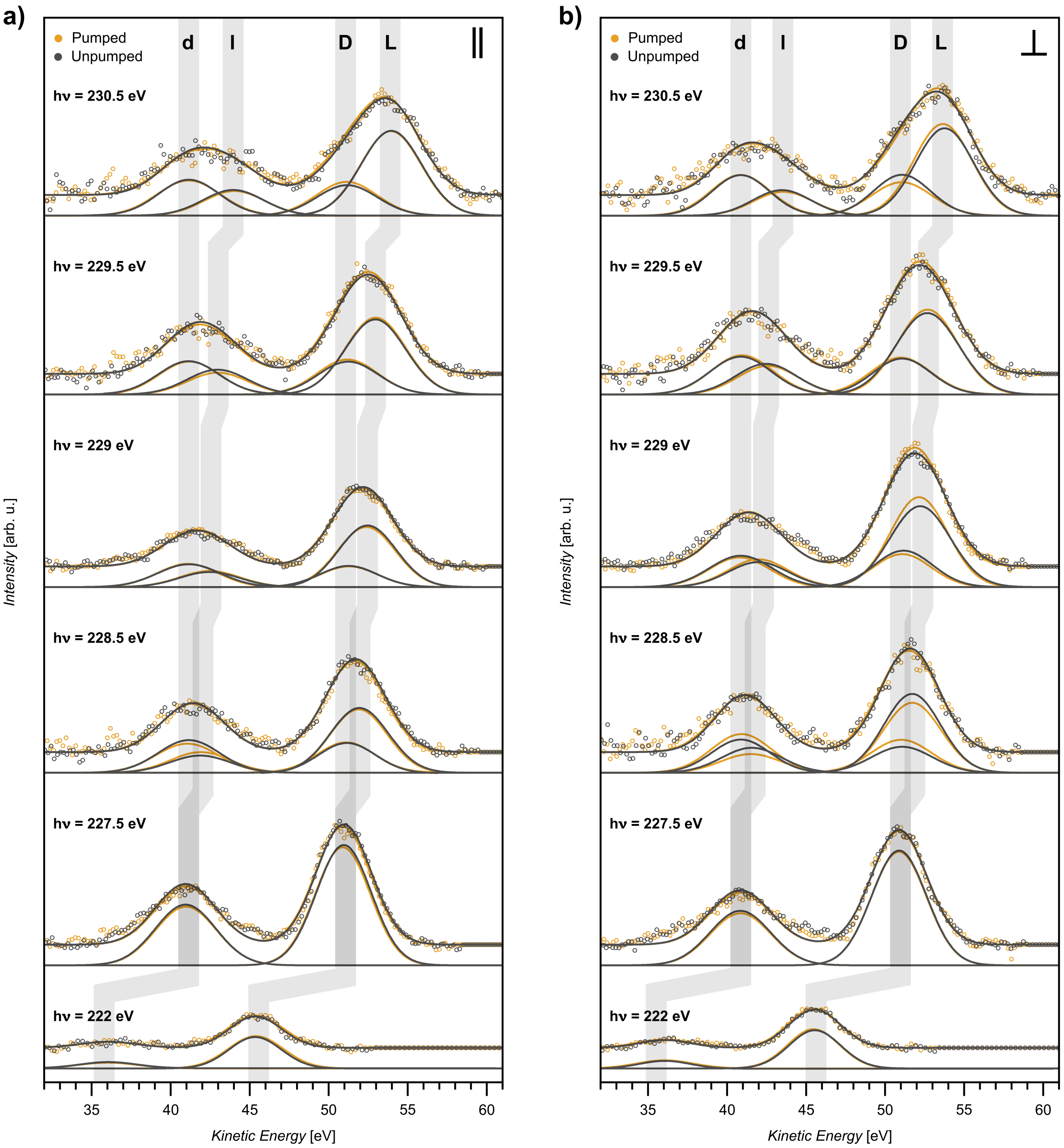

Figure S10. Directional S $L_1L_{2,3}M_{1,2,3}$ CK autoionization spectra of MoS_2 in dependence of the photon energy and optical excitation for selective preparation of S 3p excited states in the in-plane (‖) direction (**a**) and in the out-of-plane (⊥) direction (**b**). The localized Raman-channels S 2p^{-1}3s^{-1}3p^{1} (l) and S 2p^{-1}3p^{-1}3p^{1} (L) as well as the delocalized Auger-channels S 2p^{-1}3s^{-1}deloc.[1] (d) and S 2p^{-1}3p^{-1}deloc.[1] (D) are indicated. The spectral region of the direct S 2p photoionization has been removed. For comparability, the spectra are shifted according to the photoexcitation-induced shift of the S 2p core-levels.

Binding energy calibration

All XPS spectra are calibrated using a polycrystalline gold reference sample. The Fermi level (E_F) was determined by fitting the Au valence band edge (Figure S11**a**) with a Sigmoid function. All binding energies (E_B) are referred to the Fermi level ($E_B = 0\,eV$ at E_F). The calibration was verified by means of the Au $4f_{5/2}$ ($E_B = 87.4\,eV$) and the Au $4f_{7/2}$ ($E_B = 83.7\,eV$) shallow core-level doublet (Figure S11**b**). For the fitting, a Voigt curve for each of the spin-orbit split Au 4f core-levels was used after subtraction of a Shirley background[4].

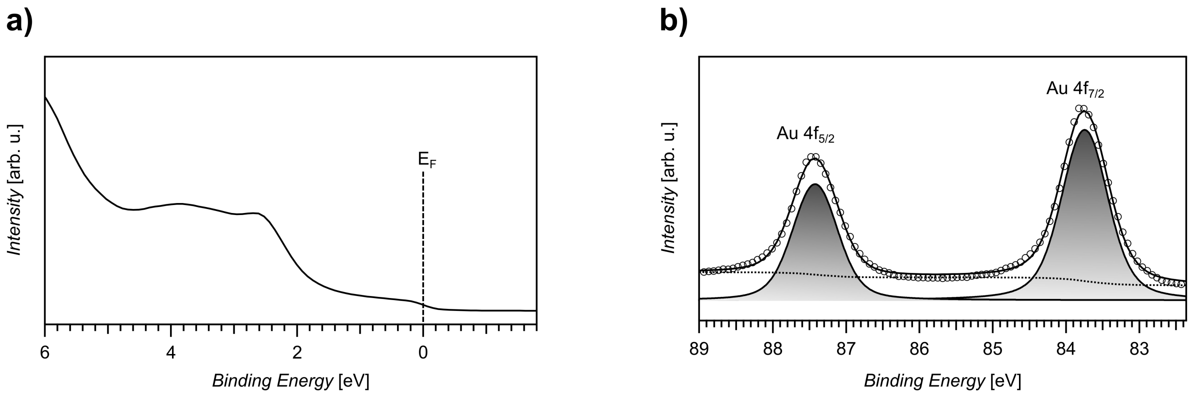

Figure S11. XPS reference measurements of the Au valence band (**a**) and the Au 4f core-levels (**b**) for binding energy calibration. The fit (black curve) to the Au 4f spin-orbit split doublet is obtained by a Shirley background subtraction (dashed line) and respectively a Voigt curve for the Au $4f_{5/2}$ and the Au $4f_{7/2}$ core-level.

Temporal overlap determination

The precise delay time zero, i.e. the time the 400 nm optical pulse arrives coincidentally with the probing X-ray pulse, is determined by means of the space-charge effects on the shallow Au 4f core-levels. Intense optical excitation of a polycrystalline gold surface emits a high number of conduction electrons into the vacuum and produces an electron cloud in front of the sample surface. Coulomb interaction of the conduction electron cloud with the Au 4f photoelectrons leads to a kinetic energy shift of the photoelectrons emitted before (negative delay times) and after (positive delay times) the creation of the electron cloud potential. The maximum of this space-charge induced Coulomb repulsion and acceleration of the photoelectrons occurs for the simultaneous emission of the conduction electron cloud and the photoelectrons, i.e. the delay time zero[5]. The dynamics of the Au 4f core-electrons under the influence of a space-charge potential, produced by intense laser pulses is shown in Figure S12.

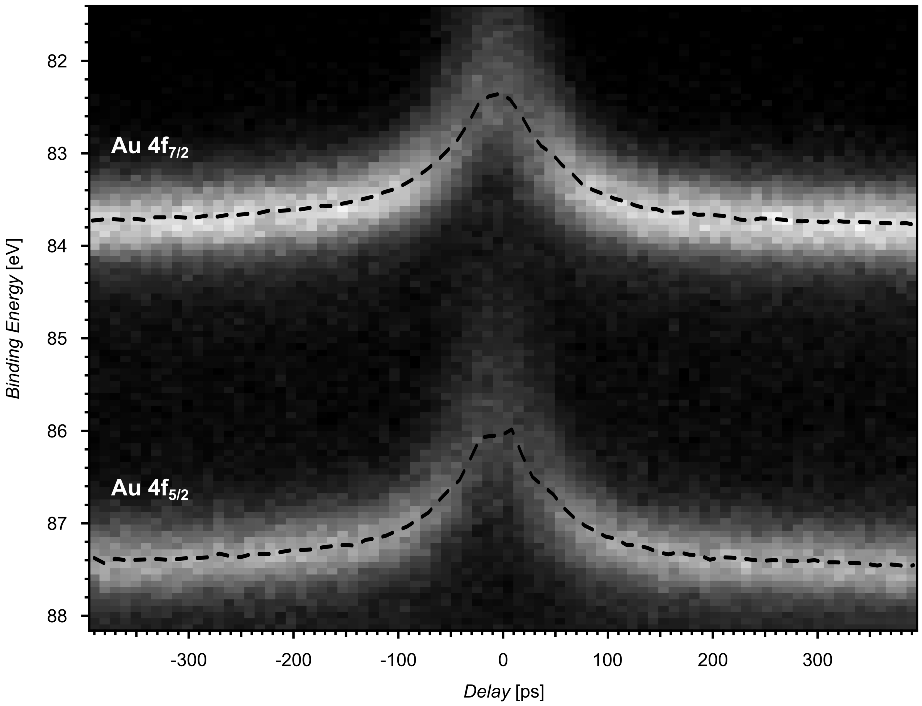

Figure S12. Two-dimensional intensity plot of the Au 4f core-level spectra as a function of the delay time between the 400 nm optical pulse and the X-ray pulse. The dynamics are recorded from -400 ps to +400 ps in 10 ps steps with a Laser fluence of 11.3 mJ/cm^2. The position of the Au 4f$_{5/2}$ and the Au 4f$_{7/2}$ core-level (dashed lines) are derived from independent Gaussian functions.

XPS sample characterization

To verify the sample quality and absence of contamination, an initial XPS survey characterization has been conducted. Figure S13 shows exemplary XPS survey spectra of 1T-TaS_2 (**a**) and 2H-MoS_2 (**b**) recorded at a photon energy of $h\nu = 800\,\text{eV}$. For both, 1T-TaS_2 and 2H-MoS_2, all observed spectral features can be assigned to the Ta and S core-levels, respectively Mo and S core-levels along with their energy loss features. No traces of O, N or C species are detectable and confirm an atomically clean sample surface.

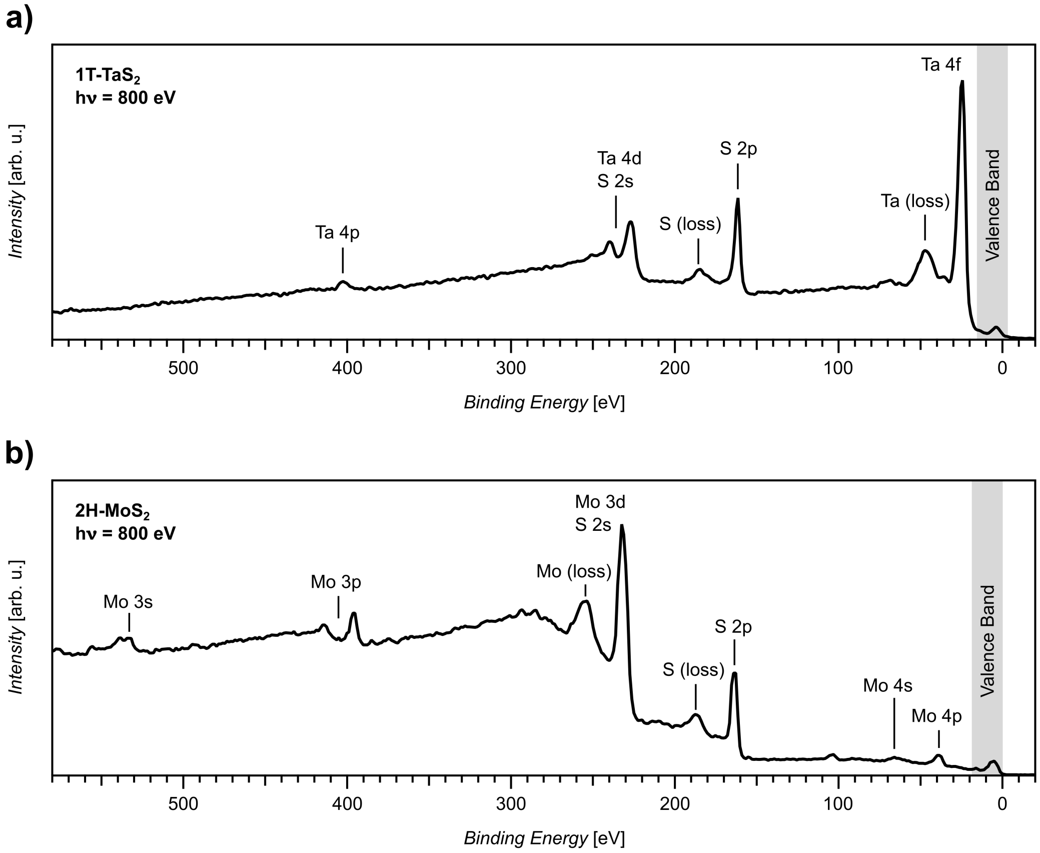

Figure S13. Exemplary long-range XPS survey characterization of 1T-TaS_2 (**a**) and 2H-MoS_2 (**b**), recorded at a photon energy of $h\nu = 800\,\text{eV}$.

References

1. Doniach, S. & Šunjić, M. Many-electron singularity in X-ray photoemission and X-ray line spectra from metals. *J. Phys. C: Solid State Phys.* **3**, 285–291, DOI: 10.1088/0022-3719/3/2/010 (1970).

2. Papageorgopoulos, C. A. & Jaegermann, W. Li intercalation across and along the van der Waals surfaces of MoS_2 (0001). *Surf. Sci.* **338**, 83–93, DOI: 10.1016/0039-6028(95)00544-7 (1995).

3. Sorgenfrei, N. L. *et al.* Photodriven Transient Picosecond Top-Layer Semiconductor to Metal Phase-Transition in p-Doped Molybdenum Disulfide. *Adv. Mater.* **33**, 2006957, DOI: 10.1002/adma.202006957 (2021).

4. Shirley, D. A. High-Resolution X-Ray Photoemission Spectrum of the Valence Bands of Gold. *Phys. Rev. B* **5**, 4709–4714, DOI: 10.1103/PhysRevB.5.4709 (1972).

5. Kühn, D., Giangrisostomi, E., Jay, R. M., Sorgenfrei, F. & Föhlisch, A. The influence of x-ray pulse length on space-charge effects in optical pump/x-ray probe photoemission. *New J. Phys.* **21**, 073042, DOI: 10.1088/1367-2630/ab2f5c (2019).
